# Home care in Europe: a systematic literature review

**DOI:** 10.1186/1472-6963-11-207

**Published:** 2011-08-30

**Authors:** Nadine Genet, Wienke GW Boerma, Dionne S Kringos, Ans Bouman, Anneke L Francke, Cecilia Fagerström, Maria Gabriella Melchiorre, Cosetta Greco, Walter Devillé

**Affiliations:** 1 NIVEL-Netherlands Institute for Health Services Research, Utrecht, the Netherlands; 2 Department of Health, Organization, Policy and Economics, Faculty of Health, Medicine and Life Sciences, Caphri, Maastricht University, Maastricht, the Netherlands; 3 Department of Public and Occupational Health, EMGO Institute for Health and Care Research (EMGO+) of VU University Medical Center, Amsterdam, The Netherlands; 4 School of Health Sciences, Blekinge Institute of Technology, Karlskrona, Sweden; 5 INRCA - National Institute of Health and Science on Aging, Ancona, Italy; 6 Faculty of Social and Behavioural Sciences, Department of Sociology and Anthropology, University of Amsterdam, Amsterdam, the Netherlands

**Keywords:** home care, European Union, care systems, international comparison

## Abstract

**Background:**

Health and social services provided at home are becoming increasingly important. Hence, there is a need for information on home care in Europe. The objective of this literature review was to respond to this need by systematically describing what has been reported on home care in Europe in the scientific literature over the past decade.

**Methods:**

A systematic literature search was performed for papers on home care published in English, using the following data bases: Cinahl, the Cochrane Library, Embase, Medline, PsycINFO, Sociological Abstracts, Social Services Abstracts, and Social Care Online. Studies were only included if they complied with the definition of home care, were published between January 1998 and October 2009, and dealt with at least one of the 31 specified countries. Clinical interventions, instrument developments, local projects and reviews were excluded. The data extracted included: the characteristics of the study and aspects of home care 'policy & regulation', 'financing', 'organisation & service delivery', and 'clients & informal carers'.

**Results:**

Seventy-four out of 5,133 potentially relevant studies met the inclusion criteria, providing information on 18 countries. Many focused on the characteristics of home care recipients and on the organisation of home care. Geographical inequalities, market forces, quality and integration of services were also among the issues frequently discussed.

**Conclusions:**

Home care systems appeared to differ both between and within countries. The papers included, however, provided only a limited picture of home care. Many studies only focused on one aspect of the home care system and international comparative studies were rare. Furthermore, little information emerged on home care financing and on home care in general in Eastern Europe. This review clearly shows the need for more scientific publications on home care, especially studies comparing countries. A comprehensive and more complete insight into the state of home care in Europe requires the gathering of information using a uniform framework and methodology.

## Background

Current demographic developments in Europe have resulted in increased interest in home care. The share of the population aged 65 years and over is increasing [1] and more people will consequently be care-dependent in the near future. Changing life-style trends [2], smaller families [3] and growing labour market participation of women have reduced the possibilities of providing care informally [2]. Growing demand for care, in combination with the diminished potential for informal care, is likely to result in a need to expand formal care services and increase expenditure. Several European countries aim to stimulate community living and care, including home care [4], a concept which is not only regarded as just a potentially cost effective way of maintaining people's independence, but is also the mode of care preferred by clients. "Home is a place of emotional and physical associations, memories and comfort", as reported by the World Health Organisation [5].

The European Commission has prioritised the gathering of information on this sector (EC Work Plan 2006 of the Programme of Community Action in the field of Public Health). Information of this kind is intended to help decision makers to develop a response to the expected rising demand for health and social services provided at home and the EC has consequently funded the EURHOMAP study ('Mapping professional home care in Europe' [6]), which is implemented by an international consortium of nine institutes from nine European countries.

As a first step in describing home care in Europe, the EURHOMAP project has undertaken a systematic review of the scientific literature, with the aim of finding out what the scientific literature in the past decade had to say about home care in European countries. Home care was defined for this review as 'professional care provided at home to adult people with formally assessed needs', which includes rehabilitative, supportive and technical nursing care, domestic aid and personal care, as well as respite care provided to informal caregivers. Home care can range from care for persons with complex needs (for instance 24 hours support) to care for those who only need help occasionally with relatively simple tasks, e.g. domestic aid for frail elderly people and adults with a handicap. Both long-term care and short-term care, for instance for patients after hospital dismissal, were included.

This review aimed to answer the following question: *'What is known in the scientific literature about home care in Europe'? *This article starts by presenting the research methods, including a detailed description of search and selection criteria. In the results section, the study characteristics will first be set out, i.e. the countries covered and the aspects of home care studied. Secondly, the depth and focus of information available per country will be discussed and finally, the key characteristics of home care systems will be presented. In the discussion section the main differences in home care within and between countries will be considered and information gaps will be identified.

## Methods

### Search strategy

The following electronic databases were searched: Cinahl, the Cochrane Library, Embase, Medline, PsycINFO, Sociological Abstracts, Social Services Abstracts, and Social Care Online. The search was limited to papers published between January 1998 and October 2009 and to studies involving persons aged 18 years and over when possible.

The search strategy (see Additional file 1 for an example) was first devised for use in Medline and subsequently adapted for the other databases. The search term 'home care services' was used for Medline; terms associated with 'home care' were used for the title or abstract in the other databases if required (MeSH term; major focus and/or exploded depending on the database). In the case of Sociological Abstracts and Social Services Abstracts for instance, the keywords 'home care', 'home help', 'home maker' and 'domiciliary care' were used. In the case of the Social Care Online database for example, the term 'home care' was searched for as a topic or in the title and countries were searched for as a topic only (searching in abstracts was impossible and a search in the free text led to too many irrelevant hits). The search was carried out for the 31 countries covered by the EURHOMAP project, i.e. the 27 EU countries and Switzerland, Norway, Iceland and Croatia. The names of these countries were also included as search terms.

### Methods of screening and article selection criteria

An initial screening of publications, based on titles, was performed by two researchers (first AB, then NG). In the second screening round of the remaining publications, titles and abstracts were evaluated by pairs of reviewers independently (NG, DSK, WGWB, AB, WD, ALF, CF, MGM). As a final screening step, the full texts of the remaining publications were independently assessed for inclusion by pairs of reviewers once more (NG, DSK, WGWB, AB, WD, ALF, CF, MGM). Any discrepancies between reviewers were resolved through consensus and, if necessary, by consulting a third reviewer.

Papers were excluded on the basis of the following criteria:

- published in languages other than English

- not related to the countries specified

- not relevant to the study question

- not in line with the working definition

- reports of effects of specific clinical interventions

- books, reports and dissertations

- reviews (as relevant individual papers would be included)

- published before 2003 (if describing organization or financing of home care)

- studies on which more recent publications were available

- covering instrument developments (e.g. needs assessment instruments)

- covering local (unstructured) projects, personal opinions and experiences

### Data extraction

After final selection of the papers, information was extracted from the full texts. The following information was extracted from the articles that met the inclusion criteria: the study results, country, author, year of publication, study design, study population, study focus and the home care domains they covered.

The framework used to identify and categorise the features of home care was based on an international comparison previously conducted in EU Member States [7]. This study used the following framework: the context of home care; the specific organisation of home care; and aspects of financing. The 'organisation of home care' comprised the organisations that provide home care, manpower, client populations, provision of services/needs assessment, problems and recent developments, and relations between home nursing and home help services. The data gathered on financing focused on payment and insurance, funding of organisations and payment of home care professionals. This framework was adjusted after consultation with the EURHOMAP consortium (8 international experts in health services research) and taking into account the information from the studies that met our study criteria.

This resulted in the following four key domains that were used in this literature overview to organise the information: *policy & regulation *(PR)*; financing *(FI)*; organisation & service delivery *(OS)*; *and *clients & informal carers *(CI). We distinguished policy and regulation as a separate dimension from financing, organisation and delivery. The area of clients & informal carers was added, because client choice and client-centeredness have become core issues in Europe [4]. Formal acknowledgement of informal care and professional support for informal carers have recently become major policy issues [4] and the demand side (client and informal caregiver) was therefore established as a separate area.

## Results

### Search flow

**Figure 1:**
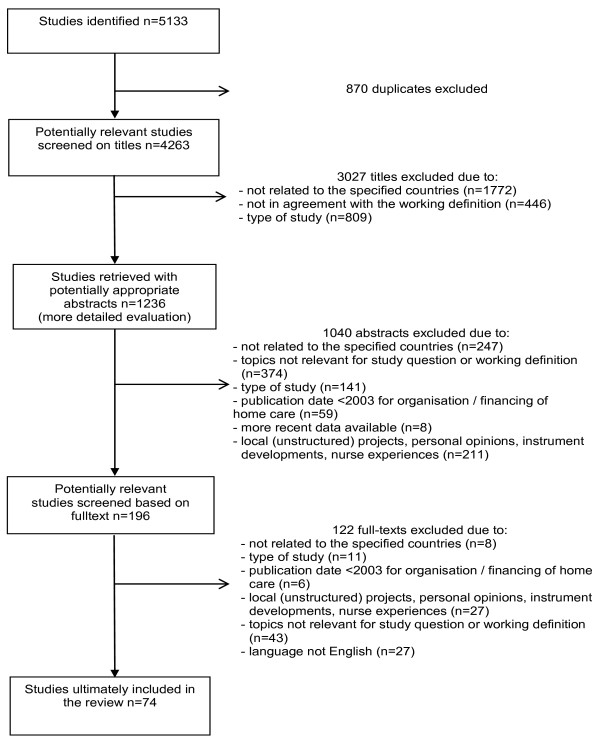
**Study selection process**.

A total of 5,133 publications were identified, 870 of which turned out to be duplicates. 4,263 were selected for further scrutiny on the basis of screening the titles. Following a review of the abstracts of 1,236, the full texts of 196 publications were retrieved and assessed on their national home care information. After the final review round based on full texts, 74 publications were finally included (see Figure 1). An overview of the studies included and their general characteristics is presented in Additional file 2.

### Study characteristics

#### Countries covered by the information

The publications included provided information on 18 countries. No information was found on Bulgaria, Croatia, Cyprus, Estonia, Greece, Hungary, Iceland, Latvia, Lithuania, Luxembourg, Malta, Romania and Slovakia. Single country studies described features of home care in 15 countries. Country comparisons were made in eight publications and these contained information on three countries for which no single country studies were found (Germany, Czech Republic and Austria). The countries addressed in the largest number of publications were Sweden and the UK. The review yielded very little information on home care in the countries situated in Central and Eastern Europe [8-10].

#### Design and population of the studies

A large majority (66) of the studies have a descriptive [9,11-28] or cross-sectional [8,10,29-73] design, while eight are prospective or retrospective cohort studies. The study populations consisted mainly of elderly persons [9,11,14,28,29,34-38,43,51,54,56,57,59,60,62-64,72-76], but home care professionals too were often the subject of study [12,17,18,21,30,32,45,46,49,53-55,58,65-68,71]. Only a few studies focused on specific types of clients (e.g. people with dementia) [9,31,32,68]. Finally, many studies had a limited geographical scope and explored a single municipality or area. This may be related to the decentralised responsibility of home care that prevails in several countries. Another, more likely, explanation is that these studies are case studies focusing on a specific service structure, and thus cannot take a broader perspective.

#### Domains and study focus

Most studies provided information on more than one of the four domains distinguished ('policy & regulation', 'financing', 'organisation & service delivery' and 'clients & informal carers') [8,9,11-16,18,19,21,23,25,26,28-31,34,35,37-41,44,47-51,55,57,58,60-62,64,67,69,71,76-81]; close to one-third focused on only one of them. For most countries information was available for each of the four domains, but the amount of information per domain differed considerably. Information was available on organisation & service delivery and on financing for almost all countries included. Information on policy and regulation, and on clients and informal carers was also widely available, but usually less extensive.

A large number of studies focused on the supply side of the home care system [8-13,15,17-20,22-25,27,30,31,33,41,45,47-50,53,55,59,61,65-68,70-72,76,77], or sought to explain the differences between receivers and non-receivers of home care [29,32,34,38-40,42,44,46,51,57,58,64,73,75,79-81]. Several studies focused on the relationship between formal home care provision and informal care [28,29,32,34,38,39,42,44,46,51,54,57,58,64,69,73,75,79-81]. Quality and improvement of quality were the subject of seven studies [10,28,49,54,68-70]. When the study goals are considered within a structural framework of input (human resources, financing and regulation) - process (organisation and delivery) **- **output, it becomes clear that many studies dealt with output and outcome [8-10,19,22,28,32,34-36,38,39,42,50-54,56,57,59,60,62,64,68-70,72,73,75,78-80]. The outputs and outcomes studied included which persons received home care, effects of received home care and the (perceived) quality of services.

### Summary of the information on home care per country

Home care described by country, and the four domains, i.e. 'policy & regulation'; 'financing'; 'organisation & service delivery'; and 'clients & informal carers' can be found in Additional file 3. Cross-country comparison was not possible, however, because the information originated from different publications and was based on different study methods. Although information was available on all four domains for many countries, the amount of information differed considerably. In this section, first the available information per country is briefly discussed. Although possibly out of date, the main characteristics of the home care systems given in the literature are described. In the next section - 'Key characteristics of home care systems' - the four domains are discussed separately, also based on the information presented in Additional file 3.

#### Austria (1 article)[22]

Information (of a limited nature) was only available on the possibility of using cash benefits for home care services.

#### Belgium (4 articles) [29-32]

All four domains were covered. Information on funding related mainly to nursing care at home and only scarce information was available on policy and regulation. More information was available on the organisation of home care. Provision of home care was in the hands of competing private agencies, nurses and other home care workers. The overall financing and organisation of home care was a shared responsibility of the Belgian Federal Government and the communities of Flanders and Wallonia.

#### Czech Republic (1 article) [10]

The only source contained very little information on home care, and concerned only one geographical area. The available information concerns the quality of services provided in the home situation, which was reported to be relatively poor.

#### Denmark (4 articles) [15,27,33,34]

Many details were available on policy and regulation and on organisation and delivery, but little on financing. The decentralised home care system was highly regulated and funded from taxes. Many municipalities provided integrated home help and home nursing.

#### Finland (7 articles)[24,26,36-40]

The four domains were covered. Little basic information on the financing of home care was available, but more was reported on the recipients of home care. Regulation and provision of home care services were decentralised. Provision was mainly public, although private home care provision was stimulated.

#### France (4 articles)[23,25,35,76]

The articles offered information on each of the four domains. Basic system characteristics were described, such as: types of providers; main financing mechanisms; types and numbers of home care recipients. The information mainly referred to home care for elderly people, which used to be a strong public responsibility, but had become a sector where competition was growing. Although services were mainly financed through the national long-term care insurance, decentralisation of financing was developing and means-tested client co-payments were commonplace.

#### Germany (4 articles) [10,22,23,25]

Information on Germany resulted from international comparative studies, which focused primarily on organisation and service delivery, and on policy and regulation. Hardly any information was available on recipients and informal carers. Home care was provided by a mix of non-profit, for-profit and religion-based agencies. Competitive elements had been introduced. Home care was a universal benefit, mainly financed through long-term care insurance controlled by quasi-public health insurance funds.

#### Ireland (4 articles) [11,12,24,41]

It was possible to retrieve a great deal of information on policy and regulation, and organisation and service delivery, but there were no papers dealing with home care clients. A major characteristic of the home care system was its split provision by public providers on the one hand and highly deregulated private providers on the other hand. Private provision was stimulated through cash-for-care schemes and competitive tendering.

#### Italy (4 articles) [10,23,42,72]

The articles provided little information on home care, particularly where policy and regulation and financing were concerned. What became evident is that home care in Italy was highly decentralised.

#### The Netherlands (8 articles) [22,24,43-47,81]

The four domains were covered, but most information was on needs assessment and client characteristics. Needs were assessed in an objective and integrated way through an independent agency, with home care clients usually receiving a mix of different types of home care services. Home care was financed through national social insurance and client co-payments. Very little information could be retrieved on policy and regulation.

#### Norway (3 articles) [13,48,49]

Hardly any information was available on financing. Information on client characteristics was limited to personal assistance users (a service additional to ordinary home care). As in other Nordic countries, home care was largely decentralised. Priority was given to quality control, and a consumerist approach was pursued through care vouchers and competitive tenders.

#### Poland (1 article) [8]

The only available paper focused on home nursing in one rural area and did not contain information on financing. Home nursing is provided by family practice nurses or (more and more) self-employed nurses.

#### Portugal (1 article) [50]

Information on clients and informal carers was lacking in this article. A major objective of Portuguese policy was to maintain the autonomy of elderly people at home and to integrate the provision of care at home.

#### Slovenia (1 article) [9]

The only information available was about home nursing, or 'home health assistance' as it was called. Home health assistance was not available all over the country and was allegedly not affordable for most elderly people.

#### Spain (2 articles) [51,23]

Information was scarce, but covered the four domains. Home care was split between home health care (provided by regions) and 'Personal Community Care Service' (provided and financed by regions and municipalities) and characterised by a lack of financial resources. Unmet needs for home care of elderly people were associated with low income, low educational attainment and living alone.

#### Sweden (20 articles) [14,16,17,23,28,52-62,74,75,79,80]

Much information was retrieved. Home care was provided through two programmes: 'Primary Health Care' (often provided by county councils) and 'Home Help' (by municipalities). Home help was very comprehensive and was a universal service, although it has recently become more targeted at people with a higher dependency. Relieving the burden of informal caregivers was a policy priority.

#### Switzerland (2 articles) [78,77]

The two studies contained a modest amount of information, focusing on acute care and on one region, the Canton of Vaud. Financing and organisation of home care (for all ages) was partly decentralised to the Cantons and communities and provided by home care agencies and home health agencies.

#### United Kingdom (16 articles) [18-21,23-25,63-71]

Most information was available on home care in the UK. Key elements of policy were client-tailored care and consumer choice. In England, provision was mainly statutorily regulated. Provision is now done by a mix of statutory, private and voluntary (non-profit) organisations. Public funding for home care came from taxation. The organisation and - in the case of home help - the setting of eligibility criteria too was largely decentralised.

### Key characteristics of home care systems

Many aspects of home care were described in the literature. The key characteristics referred to will be presented below on each of the four domains.

#### Policy & Regulation

Major characteristics of home care policy and regulation that emerged from the studies included in the review are: home care as a priority; the division between local, regional and national responsibilities; health care versus social care policy; regulation of home care benefits; regulation of the quality of services; increasing user choice; competition and co-governance; regulation of the private home care sector; and task descriptions for home care professionals. These topics are explained in more detail below.

- *Prioritising home care*. Countries differ in the extent to which they have developed an explicit policy objective on home care. Policies often included a vision that elderly people should be supported to continue living at home as long as possible. Less prominence seemed to be given to home care as a substitute for institutional care in nursing homes and hospital care.

- *Local, regional and national responsibilities*. Countries seem to differ in the allocation of responsibilities for policy, financing and delivery of services. In Finland for instance, the state regulated which welfare services needed to be in place, while the municipalities were responsible for the organisation and provision of the services [37]. In Switzerland, the health insurance co-funding home care was a responsibility of the national government, while other financial resources and policy on other issues were allocated to the Cantons [78]. In general, policy on home care was often a national affair, while the organisation and service provision were often decentralised.

- *Health care versus social care*. Policy and regulation differ according to the type of service. In Sweden, the counties were usually responsible for the organisation of home nursing, while the municipalities were responsible for home help services [55]. In Spain, the main responsibility for policy on home nursing rested with the regional governments, while on home help it was shared between municipalities and regions [51]. This sort of division of responsibilities also existed in Finland [37] and Portugal [50].

- *Regulation of home care benefits*. Allocation of home care services was guided by a set of eligibility criteria in several regions and countries, e.g. the Belgian region Flanders [29], Denmark [15], Finland [37] and the Netherlands [44]. The criteria were applied in a personal needs assessment procedure [23], possibly taking into account the financial situation and the availability of informal care [23]. Furthermore, formalisation of the needs assessment process differs [23] and seems to be stricter in France than in the UK and Sweden. In Spain and Italy, the public resources available for home care seemed to be an important determinant in the decision to assign care and income thresholds were used to allocate home care. In the Scandinavian countries, home care benefits (with the exception of domestic aid to a certain extent) are often universal, i.e. independent of income, and services are more comprehensive. National directives in the Netherlands set out the type of services that informal carers are supposed to provide [24], and in Sweden, a spouse's ability to provide care is taken into account. Age was used as a criterion in certain programmes, in addition to financial means and availability of informal care. In several countries, such as Finland [26] and Sweden [79], home care appeared to have become more targeted on those with a high level of needs. Furthermore, there were differences in the eligibility criteria for social care and home health care. In Spain and Norway for example, domestic aid was means-tested or dependent upon available informal care.

- *Regulation of quality*. Several countries have introduced regulation of or policy on quality and client-centeredness. In Norway, the national government tries to encourage quality improvements at a municipal level [49]. In the United Kingdom, the development of a skilled workforce was declared to be essential to the quality of social care, leading the government to develop organisations to stimulate and monitor the quality of home care professionals [71]. Portugal [50] and England promote training of care providers. In Poland [8], strict educational requirements have been set for home nursing providers.

- *Increasing user choice*. Policies to increase user choice were discussed in several studies. Municipalities in Denmark were obliged to give clients the choice of a private provider [15]. In Norway, personal assistance was introduced for severely disabled adults to empower them as 'consumers'; in municipalities the provider and purchaser/assessment role was split; and voucher arrangements and competitive tendering were introduced [13]. Cash-for-care programmes were also developed in the Netherlands, Finland, Ireland and England [24]. It should be noted, however, that increasing user choice and service flexibility were not the main objectives in all countries [24]. Across Europe, consumer-led approaches often seemed to be combined with service packages managed by providers and professionals, and with pooled funding [22].

- *Competition and co-governance*. Competitive elements existed in several countries, e.g. in France, Germany and Britain [25]. In France, counties were given the option of rejecting the prices set at national level, in order to increase competition. Furthermore, competitive tendering was introduced in several other countries (e.g. the UK, Ireland and Norway). Although co-governance of government and providers was still important, this was increasingly replaced by market forces [25]. Market mechanisms had weakened traditional network relations based on consensus in some countries [25], but this effect differed across countries [25].

- *Regulation of the private home care sector*. The lack of regulation of private providers was an issue in Ireland and the UK. In Ireland, the private home care sector was said to be poorly regulated compared to the public sector, resulting in quality differences and inequalities in the financing of home care providers and the working conditions of home care workers [41]. This was partially true for England as well [70]. In contrast, municipalities in Finland were responsible for the quality of all home care services, including those provided by publicly funded private providers. Complaints from recipients of privately provided care under the voucher system were filed at municipality level [24].

- *Task descriptions of home care professionals*. Nurses' tasks were officially established by a federal insurance institute at national level in Belgium [30] and by ministerial decree in Poland [8]. In contrast, private providers of home help in Ireland [12] were free to decide which tasks were to be carried out by which professional, even if the organisations were publicly funded.

#### Financing

The mode of financing differs within and between countries, as well as between home health care and home help. The following characteristics of financing emerged from the literature: the sources of funding; co-payments; allocating budgets to providers; cash for care programmes and level of expenditure on home care. These characteristics will be explained below.

- *Public sources of funding*. Home care was usually funded from a mix of sources, such as general taxation, regional and local budgets, social insurance, and private payments. In some countries, public funding came through compulsory insurance e.g. home health care in the Netherlands [44] and in Switzerland [77], in others taxes, e.g. for the home health sector in Denmark [15] and in Portugal [50]. In Spain, coverage of personal care by community services was very low [51] and private resources were required as a consequence, in contrast to the situation in, for example, Denmark [15]. The level at which funding was collected also differed. Formal care for elderly people was nationally funded in France [35], while home health agencies in Switzerland were funded by several administrative levels (federal, national and local) [77]. Funding mostly seemed to be allocated for specific types of home care services rather than for home care in general.

- *Co-payments*. Co-payments for some home care services were used in many countries, e.g. Finland, France, Ireland, England, Denmark, the Netherlands and Sweden. In most countries, the amount of the co-payments was related to the income or financial assets of the recipient (e.g. France and the Netherlands), possibly subject to a maximum [24]. Client co-payments were only needed for certain services in some countries, e.g. for home help in Sweden and only for specific services in Denmark. In some countries, e.g. Ireland [11] and Sweden [80], co-payment levels also differed between municipalities or between other lower-level authorities.

- *Allocating budgets to providers*. These budgets could be a fixed amount per day (in Belgium [30]) or payment per home care package delivered (in Ireland [12]). Different kinds of providers could be subject to different funding schemes, which could result in different incentives and unequal competition (e.g. in Ireland [12]).

- *Cash-for-care*. Payments or vouchers for recipients to buy care instead of benefits-in-kind seemed to be an important development in home care. Although such 'cash-for-care' arrangements fitted the home care system in a country, there were still some differences with the traditional manner of organising home care in that country, which resulted in increasing importance of private providers for example [24]. Cash-for-care arrangements were available in France, Germany, Sweden, England, Italy, Spain and Austria [22] and in Finland, the Netherlands and Ireland [24]. Cash-for-care schemes were introduced for several reasons, i.e. to give clients more flexibility and to tailor services more to their needs; to promote efficiency and increase competition among private providers; and to stimulate home care in general [24]. The relative importance of cash-for-care differed between countries [24], as did the eligibility criteria, prevailing quality control measures and whether the schemes were meant to replace or complement traditional care [22]. In France [35], the schemes replaced benefits in kind, while in Ireland [24] they were meant to complement these. Quality control was minimal in Ireland, in contrast to the Netherlands and Finland, where there were a range of quality mechanisms [24]. Decisions on the level of cash benefits were part of the regular needs assessment procedure in Sweden, while in Germany, Spain, Italy, France and the UK it was a separate procedure [23]. Problems reported in connection with cash-for-care programmes were the lack of regulation and coverage of costs; barriers to taking up the budget (such as lack of information among users); professionals wanting to control funding; obstacles to their use by people with a cognitive impairment [24]; and lack of support for cash benefit holders [22].

- *Adequacy of home care expenditure*. The level and adequacy of public expenditure on home care was discussed in many articles. Funding shortages were reported in Spain [51] and Portugal [50], assignment of care in Italy seemed to depend on the financial resources available [23], and home health assistance seemed not to be affordable to most elderly people in Slovenia [9].

#### Organisation & Service Delivery

The following key aspects of organisation and service delivery in a home care system were identified in the literature.

- *The type of home care providers*. A variety of provision models was found, including monopolist agencies providing comprehensive services in an area; agencies for specific services, such as nursing or domestic care (e.g. in Sweden [55]); competing commercial and non-commercial private providers and public providers. Private provision (including non-profit) was growing in several countries, such as Ireland [11], Finland [26], Sweden [74] and England [70], either replacing public provision or compensating for its absence. The introduction of market mechanisms in some countries appeared to have weakened co-governance between the third sector (voluntary sector) and the public sector [25]. The for-profit private providers may have been better adapted to the new market forces than the voluntary organisations, as was the case in the UK, where managers of voluntary organisations were more likely to have greater problems with negotiating contracts than private provider managers [18].

- *Home care integration with other types of services*. Integrated provision of services was reported to be a major challenge in some articles, e.g. in Portugal [50] and for personal budget holders in the Netherlands [24]. Integration problems are: poor service coordination as too many professionals are involved in caring for one client; multiple entry points for those seeking home care; and different jurisdictions and budgets applicable to health and social home care. Integration could be achieved by having different disciplines working within one agency and by the use of case managers. Case managers for the coordination of home care services were reported in five countries out of 11, i.e. England, Iceland, Sweden, Italy and Finland [76]. Other integration methods were integrated care teams, reported from Norway [13], integrated care trusts in the UK, organisations providing multiple types of home care, such as some domiciliary support services in Portugal [50], and most Danish [33] and some Swedish [61] municipalities. Problems with integrating complementary services and regular home care services were also reported, such as different financial conditions in England and Wales with regard to intermediary care [19]. Another issue is the coordination between home care and other services. Coordination between hospital and home care is an issue in the UK [21], where intermediary care (home care as well as residential care) has been introduced to speed up hospital discharge and to prevent unnecessary re-admissions. In Finland, home helps also delivered care in residential care units and assisted living arrangements [26]. In Poland, home nurses were often employed by family doctors [8], thus becoming part of the primary health care system.

- *Accessibility of home care*. In Sweden, geographical variation in access to home care was related to different needs across regions [49]. It is possible that such differences are also related to differences in available resources between regions, as is the case in Spain [51] and Slovenia [9]. Variation in access may also be related to the absence of formalised needs assessment instruments [23]. Assessment was more formalised in France than in the UK and Sweden, where assessors had wider discretionary powers. In Italy and Spain, assessment depended on the region and the assessment team. Lack of standardisation of assessment was also a point of concern in the Netherlands [44]. In general, countries differed in the formalisation of the procedure, the instruments used, the professionals involved, and whether social needs were taken into account in addition to physical needs [23]. The organisations performing the assessment could be independent assessment agencies (the Netherlands [24]), municipal teams independent of provision (Norway [13]), or governmental organisations (the local social service departments in the UK and the municipal care teams in Finland [24]). In France, Germany, the UK, Sweden and Spain, needs assessment was followed by the preparation of a care plan that included the services to be provided and the number of hours [24]. In two countries with public provision, Sweden [79] and Finland [26], a shift in focus over time was reported from low level needs to those with the highest level of needs.

- *Monitoring of care needs*. Several studies mentioned monitoring and reassessment of clients' needs after a period of time. In Sweden, care was only assigned for a few months and was regularly monitored [23]. In the UK, care provided was examined for adequacy after 6 weeks [20] and care plans were adapted every 6 months, while needs were re-assessed after 6 months in Finland [24].

- *Quality of home care*. The quality of home care was discussed for several countries, such as Norway [49], UK [68] and Sweden [52]. Reported instruments for quality improvement were: strict supervision; use of protocols; and user surveys. An international comparison, restricted to urban sites in 11 countries, showed the quality of home care to be most problematic in the Czech Republic and Italy, and least problematic in the Nordic countries [76]. Satisfaction surveys were used by almost two-thirds of the municipalities in Norway. Quality improvement initiatives in Norway were generally not focused on technical quality [49].

- *Working conditions for home care workers*. Working conditions were also discussed in several papers. A study in Northern Ireland [67] showed that home care workers were dissatisfied with irregular working hours, lack of management support and workload pressures [66]. Burn-outs were reported among home care workers in the Netherlands [45]. The position of workers in the private sector was weaker than in the public sector in Ireland, in terms of payment, working conditions and qualifications [11,41].

- *Increasing self-care ability*. 'Re-ablement' programmes were set up in the UK, with the objective of enhancing self-care among dependent people and hence empowering them to live at home. Municipalities in Denmark were legally obliged to carry out preventive home visits to citizens over the age of 75 [34] with the aim of fostering the functional abilities of these citizens and improving the use of their own resources [34]. In a Polish rural area, most home visits by family nurses were devoted to health education [8].

#### Clients & informal carers

Where clients, informal carers and client empowerment were concerned, the following domains were identified in the studies included.

*-Elderly people covered by home care*. In a number of countries, substantial proportions of the elderly population received home care. In France, for instance, over one-third of people over 75 received home care [35]. In Finland, however, only 6.3% of people over 65 received home care regularly in 2003 [39], while almost a quarter of the total population of a rural area in Poland was visited by a home nurse. In Denmark, 60% of those over 75 received preventive home visits [34].

- *Characteristics of home care recipients*. Advanced age, being female [34], higher educational attainment in some countries and lower educational attainment in other countries [40,51,56,64], and the recipient's income [51] were reported as being related to the receipt of home care. Furthermore, the use of home care in some countries appeared to relate to functional disabilities and general and specific psychological characteristics, e.g. depressive moods in Belgium [29], Spain (Madrid area) [51] and Sweden (Stockholm) [75], and the general psychological characteristics of men who received preventive visits in Denmark. Frail elderly people are a prominent client group in home care. Many studies indicated the importance of cohabitation status or having or not having a spouse, in relation to receiving home care. In Belgium [29], Sweden (Stockholm) [57,75] and Finland [39], those living alone received more home care than those cohabiting, while in France [35] people with a spouse were more likely to receive formal home care. In Spain (Madrid area) [51], living alone was associated with unmet needs.

- *Relationship between informal and formal care*. Relieving informal carers by offering professional care was mentioned as a policy objective in several countries, e.g. Portugal [50] and Sweden [60]. The presence of a spouse and, therefore, the possible availability of informal care influenced the receipt of formal home care in some countries. Care needs were met by a mix of formal and informal care in France [35], Finland [38], Italy [42] and Southern Sweden [62], where informal care could also be a substitute for formal domestic aid to some extent. An international comparison showed informal care to be a substitute for formal care in Southern Europe, but not in Central European countries [73]. The type of informal carer was also reported to make a difference in this respect. Parents in Finland were more likely to receive formal care when their children were providing informal care, possibly because the children acted as agents in applying for care [38]. Recipients' preferences played a role in the choice of formal or informal care. A study in the Netherlands reported that most elderly people preferred to receive personal care from home care professionals, while informal carers were accepted more readily for domestic assistance. The financial compensation for informal carers differed between countries [43]. In the Netherlands [24], England [24] and Austria [22], informal carers could be paid through cash-for-care programmes, and in Finland [38] and Ireland [11] through modest carer allowances (with or without income restrictions).

- *The availability of respite care*. Respite care was reported in the Netherlands [46] and in Finland [37]. A Dutch study showed that one-third of sampled informal carers received respite care [46].

## Discussion

### A limited and skewed picture

This systematic literature review has shown that the scientific literature published in English provides rather limited information on home care in Europe. Seventy-four relevant studies were traced, only a few of which compared countries. No information was available on more than one-third of the countries included in the review and the information available was quite unevenly distributed across countries. Information on Central and Eastern European countries was particularly scarce. One-third of the studies focused on just one of our study domains (organisation & service delivery; financing; clients & informal carers; policy and regulation), which meant that the home care context (other facets of the home care system) was not always described. Furthermore, most studies were small-scaled and the degree of detail in the information differed considerably. Most was reported on the domains of client characteristics (as predictors of the use of home care), the organisation and delivery of home care services. Little detail was provided on the financing of home care. Many studies focused mainly on elderly people. In sum, the information from the scientific literature does not permit a full comprehension of the core aspects of home care in European countries.

### Variations across Europe

Our study has pointed to international differences in policies on home care, in the practical organisation of home care and in the availability of services. With regard to policy and regulation, a number of countries had set criteria for eligibility; while several took the financial situation and availability of informal care into account, others did not do so. Countries targeted different population groups with their home care systems. In several Mediterranean countries, governments focussed on the poorer population, while there seemed to be no targeting of this kind in other countries. Many countries had decentralised some of the responsibilities for policy development, financing and organisation to local and regional governments. The articles pointed to an integrated vision and policy on care and cure at home in some countries, while in others policies on home health and social services were separate.

On the subject of financing, the articles mainly focused on funding mechanisms and shortages. A contrast in the level of public funding existed between Scandinavian countries, on the one hand and Spain and Portugal on the other (respectively high and low). However, in Sweden and Finland a new tendency was to increasingly concentrate on the core - clients in the greatest need of care - and less on Instrumental Activities of Daily Living services. Although funding mechanisms differed across countries, a mix of mechanisms was often in place.

A great variety of home care providers was identified: public, private non-profit, private for-profit, or a mix of these. Their importance differed across countries. Providing agencies could either offer a range of home care services or be specialised in only one. In several countries, particularly in England, Ireland and Scandinavian countries, there was a trend of increasing (contracted) private provision. A potential problem was that regulations sometimes affected traditional players and new ones unequally, leading to differences in working conditions.

Some countries, with very different home care systems, had developed arrangements intended to support self-care. Possibly, the general financial pressures are causing the interest in such arrangements. In some countries, care provided by non-professionals was also encouraged through paying informal carers or funding respite care. A major focus was also the relationship between formal and informal care. Formal care was found to be complementary in some countries, but rather a substitute in others. Finally, several papers reported on client-centred approaches, such as cash-for-care schemes.

### Variation within countries

Like it appeared between countries, heterogeneity in regulation, financing, delivery and availability of services was also found within countries. It differed between local governments and between types of home care services. In some countries lower level authorities may set their own priorities, and develop their own financing and criteria, possibly resulting in disparities between areas in access and quality of services.

Lacking coordination and integration of services provided in the clients' home was reported in several studies. This problem prevailed in particular between home health care and social care at home. Mechanisms were reported to counteract poor coordination.

### Strengths and limitations of this review

This review has provided a systematic overview of the recent scientific knowledge on home care. It had a broader geographical scope than previous overviews of home care systems [4,7,82,83]. The focus on scientific literature was chosen to safeguard the quality of the information, but it may have been a limitation as well. Good studies may also appear as grey literature, in particular those published in other languages than English, which is the usual language of many international scientific journals. So, the conclusions of this review are not based on possible relevant publications in other languages than English. Another limitation has been that no free text search for 'home care' was carried out in the final design of the review. Such a search has been done provisionally but has been rejected because it resulted in unmanageable large numbers of hits with no or minor relevance to home care. The use of a limited number of databases for the search could be another source of missed information. To ensure coverage of the two main areas of home care, that is, social services and health care services, data bases from both areas were used; three for social services and five for health care. Given the coverage of these data bases, it is reasonable, however, to assume that major articles on home care would be retrieved with this approach.

### Further research

This review clearly showed that current information in the scientific literature is incomplete, fragmented and not well suited to make international comparisons. Nevertheless, this review has provided a feel for the variation in models of governance, financing and organisation and delivery. A comprehensive evaluation using a uniform definition and methodology is needed to obtain the full picture. On the basis of the results of this review, next phases of the EURHOMAP-project seek to systematically describe and compare home care systems in the European Union.

Domains that deserve more attention of researchers are financing; home care to other population groups than elderly people; and practical aspects of the provision of home care, reflecting the daily practice of home care professionals in their work with clients and patients. In general, research is needed to show how national level arrangements and practical models of provision are related to outcomes. In planning reforms it is crucial for governments to understand such relationships. Few publications paid attention to this relationship. Possible explanations of differences in outcomes may for example be sought in the financial resources available to countries or municipalities, the task differentiation between actors in home care, the overall extent of service provision, overarching health care systems, or even welfare state regimes. An important subject for research is coordination and integration of services in the light of models of decentralisation of social and health care services. It is evident that home care cannot be studied without taking features of the health care system into account. For instance, a Bismarckian health care context has different implications for home care than a Beveridge type of health care context. Furthermore, health care systems in general may be different than the social care system in terms of professionalisation, being more hierarchical, better funded and more rights-based [84]. This review showed that home care systems and health care systems in Europe are differently intertwined.

In the context of severe financial constraints, demographic developments and the resulting expected rising expenditures on care will urge many countries to reconsider their home care systems. International comparisons can provide decision makers with new models and innovations for home care tuned to varying public budgets in the countries of Europe.

## Conclusions

As a first step in a project to systematically describe home care in Europe, this review aimed to find out what the scientific literature in the past decade has reported about home care in European countries. The papers included in the review have provided a great deal of information, but have not been able to provide a complete picture of home care in Europe. Many studies focused on just one aspect of the home care system and little information emerged on financing. Furthermore, very few papers dealt with home care in the countries of Central and Eastern Europe. A comprehensive and balanced insight into the state of home care requires a framework applied in an international study using a uniform methodology.

## Competing interests

The authors declare that they have no competing interests.

## Authors' contributions

NG performed major parts of the review and wrote the draft manuscript. WB was reviewer and co-author of the manuscript. AB performed part of the review and reviewed drafts of the manuscript. DK and WD were reviewers and co-authors of the manuscript. ALF, CF, CG and MGM were reviewers and reviewed drafts of the manuscript. All authors read and approved the final manuscript.

## Pre-publication history

The pre-publication history for this paper can be accessed here:

http://www.biomedcentral.com/1472-6963/11/207/prepub

## Supplementary Material

Additional file 1 **Example search strategy**. Search strategy, Word, Example search strategy, A search strategy for Medline is presented as an example. Click here for file

Additional file 2 **Overview of the study characteristics**. Overview of the study characteristics, Word, Overview of the study characteristics, An overview of the general characteristics of the studies included is presented, such as the study focus and scope of the research. Click here for file

Additional file 3 **Description of home care by country and key domains**. Description of home care by country and key domain, Word, Description of home care by country and key domains, Home care is described by country and the four domains, i.e. 'policy & regulation'; 'financing'; 'organisation and service delivery'; and 'clients & informal carers' per country. Click here for file
