# Supplementary material for: Home care in Europe: a systematic literature review
Source: BMC Health Serv Res. 2011 Aug 30;11:207. doi: 10.1186/1472-6963-11-207 (PMC3170599; doi:10.1186/1472-6963-11-207)
Supplement: Additional file 1 — Example search strategy. Search strategy, Word, Example search strategy, A search strategy for Medline is presented as an example. [file 1472-6963-11-207-S1.DOC]

Additional file 1 - Example search strategy

("Austria*"[All Fields] OR "Belgi*"[All Fields] OR "Bulgaria*"[All Fields] OR "Cypr*"[All Fields] OR "Czech*"[All Fields] OR "Denmark"[All Fields] OR "Danish"[All Fields] OR "Estonia*"[All Fields] OR "Fin*"[All Fields] OR "France"[All Fields] OR "French"[All Fields] OR "German*"[All Fields] OR "Greece"[All Fields] OR "Greek"[All Fields] OR "Hungar*"[All Fields] OR "Ireland"[All Fields] OR "Irish"[All Fields] OR "Ital*"[All Fields] OR "Latvia*"[All Fields] OR "Lithuania*"[All Fields] OR "Luxembourg*"[All Fields] OR "Malt*"[All Fields] OR "Netherlands"[All Fields] OR "Dutch"[All Fields] OR "Poland"[All Fields] OR "Polish"[All Fields] OR "Romania*"[All Fields] OR "Slovakia*"[All Fields] OR "Slovenia*"[All Fields] OR "Spain"[All Fields] OR "Spanish"[All Fields] OR "Swed*"[All Fields] OR "United-Kingdom"[All Fields] OR "UK"[All Fields] OR "Britain"[All Fields] OR "British"[All Fields] OR "Engl*"[All Fields] OR "Wales"[All Fields] OR "Welsh"[All Fields] OR "Scot*"[All Fields] OR "Croatia*"[All Fields] OR "Iceland*"[All Fields] OR "Norway"[All Fields] OR "Norwegian"[All Fields] OR "Switzerland"[All Fields] OR "Swiss"[All Fields] OR "Europe"[All Fields] AND (("1998/01/01"[PDAT] : "2009/10/21"[PDAT]) AND "humans"[MeSH Terms] AND ("adult"[MeSH Terms:noexp] OR ("middle aged"[MeSH Terms] OR "aged"[MeSH Terms])))

) AND (("Home Care Services"[Mesh] OR "Home Care Services, Hospital-Based"[Mesh] OR "Home Care Agencies"[Mesh] OR "Dental Devices, Home Care"[Mesh] OR "Home Health Aides"[Mesh] OR "Respite Care"[Mesh] OR "Insurance, Long-Term Care"[Mesh] OR "Homemaker Services"[Mesh] OR "Hemodialysis, Home"[Mesh]) AND (("1998/01/01"[PDAT] : "2009/10/21"[PDAT]) AND "humans"[MeSH Terms] AND ("adult"[MeSH Terms:noexp] OR ("middle aged"[MeSH Terms] OR "aged"[MeSH Terms])))) AND (("1998/01/01"[PDAT] : "2009/10/21"[PDAT]) AND "humans"[MeSH Terms] AND ("adult"[MeSH Terms:noexp] OR ("middle aged"[MeSH Terms] OR "aged"[MeSH Terms])))
