# Supplementary material for: Home care in Europe: a systematic literature review
Source: BMC Health Serv Res. 2011 Aug 30;11:207. doi: 10.1186/1472-6963-11-207 (PMC3170599; doi:10.1186/1472-6963-11-207)
Supplement: Additional file 2 — Overview of the study characteristics. Overview of the study characteristics, Word, Overview of the study characteristics, An overview of the general characteristics of the studies included is presented, such as the study focus and scope of the research. [file 1472-6963-11-207-S2.DOC]

Additional file 2 - Overview of the study characteristics

| **Country** | **Author(s)** | **Year** | **Study design** | **Study population** | **Study focus** | **Home care domain studied*** |
| --- | --- | --- | --- | --- | --- | --- |
| **Belgium** | Roelands, Van Oyen, Depoorter et al. [29] | 2003 | Cross-sectional | 1,134 elderly people living in the community aged 65+ years in Belgium. | The relation between health and social care use and cognitive impairment and depressive mood. | PR, OS, CI |
|  | De Vlieger, Paquay, Grypdonck et al. [30] | 2005 | Cross-sectional | 501 nurses in home nursing organisations in Flanders. | Exploration of the core interventions in home care. | PR, FI, OS |
|  | Roelands, Van Oost, Depoorter, Verloo [31] | 2005 | Cross-sectional | 169 home care nurses and 1259 home care workers across Belgium | Describing some conditions for effective counselling of family carers of persons with dementia and home care professionals counselling work (informing, advising about personal interaction and support them emotionally), and explore the relationship between counselling work and attitude, self-efficacy and subjective norm. | OS, CI |
|  | Roelands, Van Oost, Depoorter [32] | 2008 | Cross-sectional | 168 family care givers of persons with dementia in one region | Examining relationship between the use of home services and psychological and social characteristics of family carers | CI |
| **Denmark** | Stuart, Weinrich [33] | 2001 | Cross-sectional | Danish policymakers; n= n.a. | The implementation of home- and community-based long-term care. | PR |
|  | Lewinter [27] | 2004 | Descriptive study | Not restricted. | The effect of administrative arrangements on the provision of home help. | OS |
|  | Stuart, Hansen [15] | 2006 | Descriptive study | Not restricted. | Description of Danish home care policy and implications for the US. | PR, FI, OS |
|  | Avlund, Vass, Lund, Yamada, Hendriksen [34] | 2008 | Cross-sectional | 5,788 non-institutionalised citizens being born in 1918, 1923 or 1924. These were selected from 34 municipalities. | Examining the relationship between psychological characteristics or social network, and the acceptance of preventative home care visits | PR, OS, CI |
| **France** | Litwin, Attias-Donfut [35] | 2009 | Cross-sectional | 631 people over the age of 75 | Examining the relationship between informal and formal care in France and Israel | PR, FI, OS, CI, |
| **Finland** | Jylhä, Hervonen [36] | 1999 | Cross-sectional | 366 persons living at home, aged 90 years or over in the city of Tampere, Finland. | To study if self-reported indicators of functioning and need for help among people aged 90 or over predict mortality and institutionalization. | CI |
|  | Kröger [26] | 2003 | Descriptive | Not restricted | Describing the developments in the Finnish care sector between 1990 and 2000. | PR, OS, CI |
|  | Söderlund [37] | 2004 | Cross-sectional | 13 elderly clients of a Finnish home service office. | The usability of ICT for home services. | PR, OS, CI |

| **Country** | **Author(s)** | **Year** | **Study design** | **Study population** | **Study focus** | **Home care domain*** |
| --- | --- | --- | --- | --- | --- | --- |
| **Finland**  **Cont.** | Blomgren, Martkainen, Martelin, Koskinen [38] | 2008 | Cross-sectional | 1,166 people between the age of 70 and 99 living in community dwellings | Estimate the frequency and determinants of use of formal and informal care by elderly persons in community dwellings . | PR, FI, OS, CI |
|  | Hammar, Rissanen, Perälä [39] | 2008 | Cross-sectional | 721 home care recipients from 22 municipalities who had had an inpatient hospital stay during the 6 months previous to survey | Describe the need, costs and use of home care and the factors associated with the receipt and costs of social and health care services. | PR, FI, OS, CI |
|  | Hammar, Perälä, Rissanen [40] | 2009 | Cross-sectional | 686 pairs of home care clients and their home care workers | Analyse association of client and municipality factors with the workers’ perception of clients’ functional ability. | PR, OS, CI |
| **Ireland** | Timonen, Doyle [11] | 2008 | Descriptive | Only restricted to Ireland, elderly people | Exploring the development of formal care for the elderly persons in Ireland | PR, OS, CI |
|  | Timonen, Doyle [41] | 2007 | Cross-sectional | 63 care workers from public, private and non-profit sectors in Ireland. | To explore the differences and commonalities between care work in the public, private and non-profit sectors. | PR, FI, OS |
|  | Doyle, Timonen [12] | 2008 | Descriptive (desk-based research and 125 interviews) | Restricted to the greater Dublin region (125 persons working in home care organisations). | To get a better understanding of the roles in delivery and financing of the public, private and non-for-profit domestic care sectors. | PR, FI, OS |
| **Italy** | Landi, Lattanzio, Gambassi, et al. [42] | 1999 | Cross-sectional | 1,354 patients admitted between 1997-98 to an integrated health care program in eleven Health Agencies in Italy. | To describe profiles of patients admitted to an integrated home care program. | PR, CI |
| **Netherlands** | Wielink, Huijsman [43] | 1999 | Cross-sectional | 2,991 elderly persons living independently, aged 65+ in the northern region of the Netherlands | The relationship between attitudes towards care and care preferences of elderly community residents. | CI |
|  | Van Campen, Woittiez[ 81] | 2003 | Retrospective cohort | 7732 applicants for home care in the northern part of the Netherlands. | To estimate the volume and composition of referrals to home care on the basis of applicant characteristics. | OS, CI |
|  | Van Campen, van Gameren [44] | 2005 | Cross-sectional | 28,047 long-term care applicants. | To find out what factors explain eligibility to several long-term care alternatives (one of which is home care). | FI, OS, CI |
|  | Xanthopoulou, Bakker [45] | 2007 | Cross-sectional | 747 Dutch employees from two home care organisations. | The relation between job demands/resources and burnout among home care personnel. | OS |
|  | Van Exel, de Graaf, Brouwer [46] | 2008 | Cross-sectional | 273 informal carers | Find associations between attitudes towards respite care, characteristics of the care giver situation and the need and use of respite care | CI |
|  | Algera, Francke, Kerkstra, Van der Zee [47] | 2003 | Cross-sectional | 78 assessment agencies, 92 home care agencies and 23 health insurers across the Netherlands | To describe the use of new style needs assessment in practice and the evaluation of it by home care agencies, assessment agencies and insurance companies. | OS, PR |

| **Country** | **Author(s)** | **Year** | **Study design** | **Study population** | **Study focus** | **Home care domain*** |
| --- | --- | --- | --- | --- | --- | --- |
| **Norway** | Askheim [48] | 1999 | Cross-sectional | 85 recipients of personal assistance (70% of the total number) | Describing the personal assistance service, its users and the user satisfaction on the service | PR, CI |
|  | Vabø [13] | 2006 | Descriptive | Not restricted. | Describing reforms in the public sector and the assumptions underlying them. | PR, OS |
|  | Kjøs, Botten, Romøren [49] | 2008 | Cross-sectional and descriptive | 32 municipalities, in each 1 home care leader and 1 nursing home leader. | To find out to what extent quality improvement activities in long-term care are implemented and what influences the implementation. | PR, FI, OS |
| **Poland** | Marcinowicz, Chlabicz, Konstantynowicz, Gugnowski [8] | 2009 | Cross-sectional | 1000 persons registered with a family doctor in 1998, and the same number in 2002 and 2006. One municipality in Northern Poland. | To describe the involvement of family nurses employed by doctors in home visits compared to self-employed family nurses, in the context of legal changes in service provision. | OS, PR, CI |
| **Portugal** | Santana, Dias, Souza, Rocha [50] | 2007 | Cross-sectional | Recipients of 75 institutions providing Domiciliary Support Services | To describe the services provided by certain home care institutions called ‘SAD’. | PR, OS, FI |
| **Slovenia** | Kogoj [9] | 2008 | Descriptive | Not restricted, focused on persons with dementia. | Describe care for persons with dementia in Slovenia. | FI, OS |
| **Spain** | Otero, García de Yébenes, Rodríguez-Laso et al. [51] | 2003 | Cross-sectional | 1,135 elderly people living in the community elderly people living in Madrid, Spain. | The prevalence of home care needs and the factors associated with unmet home care needs among community-dwelling elderly people | PR, FI, OS, CI |
| **Sweden** | Larsson, Larsson [54] | 1998 | Cross-sectional | 151 elderly home care users and their 151 caregivers in one Swedish municipality | Exploring the relationship between the perceptions of elderly home care users and their caregivers of the quality of care. | CI |
|  | Tornkvist, Gardulf, Strender [52] | 2000 | Cross-sectional | 168 adult home care and 264 outpatient clinic patients in one area of Greater Stockholm. | Patients' satisfaction with the care given by district nurses at home and at primary health care centres. | CI |
|  | Jegermalm [53] | 2002 | Cross-sectional | 43 municipalities and 208 voluntary organisations in the county of Stockholm. | To examine what support services are offered to informal caregivers and whether the support is aimed directly or indirectly at the carers. | PR |
|  | Sundstrom, Johansson, Hassin [74] | 2002 | Retrospective cohort | Swedish elderly people living in the community, in 1954, 1974, and 2000. | To describe the Swedish debate on the role of family and state in care for elderly patients. | PR |
|  | Johansson, Sundstrom, Hassing [14] | 2003 | Descriptive | Not restricted. Focused on the elderly. | To describe substitution of old-age care. | FI, OS, CI |
|  | Malmberg, Ernsth, Larsson, Zarit [55] | 2003 | Cross-sectional | 24 teams and 48 individuals active in evening and night home care patrols in the municipality of Jönköping, Sweden. | To describe the work of evening and night home care patrols in old-age care. | PR, OS |

| **Country** | **Author(s)** | **Year** | **Study design** | **Study population** | **Study focus** | **Home care domain*** |
| --- | --- | --- | --- | --- | --- | --- |
| **Sweden cont.** | Larsson, Silverstein [56] | 2004 | Cross-sectional | 390 persons over the age of 91 living in the community in an urban area in Sweden | Explore whether elderly people who live alone in community dwellings are more likely to receive informal care when they have been married and have children and whether the others are more likely to receive formal care. | CI |
|  | Meinow, Kareholt, Lagergren [57] | 2005 | Cross-sectional | 943 home help recipients aged 65 and over in an inner city district of Stockholm | Investigating the influence of client characteristics on the number of hours of home help received. | CI, OS |
|  | Olaison, Cedersund [58] | 2006 | Cross-sectional | 20 home care assessment meetings in three municipalities in Sweden. | To understand how old people, when they apply for care, describe their need for care, and how this is negotiated and positioned in assessment meetings. | PR, OS |
|  | Davey, Johansson, Malmberg, Sundstrom [59] | 2006 | Cross-sectional | 3267 individuals aged 65 and older in 2002-2003 with a coverage of public Home Help services in the 288 municipalities in Sweden. | To investigate whether reduction in old-age care services has led to inequitable service levels or simply large local variations. | OS |
|  | Larsson, Thorslund, Kareholt [75] | 2006 | Prospective cohort | 502 elderly people living in the community aged 81-100 in Stockholm, Sweden. | To identify factors that predict the use of home help services and transition into institutional care and to study to what extent the supply is client tailored. | CI |
|  | Larsson [80] | 2006 | Retrospective cohort | 2651 individuals randomly sampled from the Swedish population | Changes in time in the allocation and utilisation of public home help services and in the support and care needs of older people. | PR, FI, OS, CI |
|  | Sundström, Malmberg, Johansson [60] | 2006 | Cross-sectional (two periods) | In 2002/03 3,552 persons over the age of 65 | Analyse the balance between care provided by public home help services and family care between 1988 and 2003. | CI, PR |
|  | Janlöv, Hallberg [28] | 2006 | Descriptive | 2 municipalities | Exploring elderly peoples’ experience of participation in and influence on decisions about public home care/help during needs assessment. | CI, OS |
|  | Clevnert, Johansson [16] | 2007 | Descriptive | Not restricted. But for recipient experience section 677 recipients of assistance allowances across Sweden. | Describing the Swedish Personal Assistance Programme introduced in 1994 | PR, FI, OS |
|  | Hedman, Johansson, Rosenqvist [61] | 2007 | Cross-sectional | 211 directors of the social welfare services, or their delegates, of 211 Swedish municipalities | Studying the design and distribution of different organisational solutions regarding the responsibility for and provision of home care for the elderly in Sweden, specifically the structural integration. | PR, OS |
|  | Karlsson, Edberg, Westergren, Hallberg [62] | 2008 | Cross-sectional | 1985 persons over the age of 65 receiving long-term municipal care in Southern Sweden | Differences in the health complaints, functional ability and level of municipal care and informal care between people over 65 living at home and those living in special accommodation. | PR, CI |

| **Country** | **Author(s)** | **Year** | **Study design** | **Study population** | **Study focus** | **Home care domain*** |
| --- | --- | --- | --- | --- | --- | --- |
| **Sweden cont.** | Savla, Davey, Sundström, Zarit, Malmberg [79] | 2008 | Prospective cohort | 286 municipalities | To find out why decreased home help services have not resulted in higher score in unmet needs. | PR, OS |
|  | Petrakou [17] | 2009 | Descriptive | One Swedish county with split provision of home health care and home help. | Exploring coordination of home care workers in daily work, coordination problems and which support is needed for seamless and integrated home care for the elderly. | OS |
| **Switzerland** | Dubois, Santos-Eggimann [78] | 2001 | Prospective cohort | 95 patients taking part in the Hospital-at-Home care experiment in the Canton of Vaud, Switzerland. | Evaluation of patients’ satisfaction with hospital-at-home care. | PR, CI |
|  | Santos-Eggimann, Cirilli, Monachon [77] | 2003 | Prospective cohort | 40 home care agencies located in a Swiss region and 3816 urgent requests. | To estimate the frequency of unscheduled services delivered by home care agencies and to identify risk factors. | FI, OS, CI |
| **United Kingdom** | Hardy, Young, Wistow [63] | 1999 | Cross-sectional | 28 older service users, 20 informal carers and 22 care managers across four local authority areas in England. | To set out a typology of the choices which users and carers are expected to be able to make in the assessment and care management process. | PR |
|  | Stoddart, Whitley, Harvey, Sharp [64] | 2002 | Cross-sectional | 2,000 elderly people living in the community registered with 11 general practices in a British city. | To investigate the determinants of use of statutory and private home care services by older people living in the community. | CI |
|  | Weiner, Stewart, Hughes et al. [65] | 2002 | Cross-sectional | 101 local authority social services departments  in England. | Variation in care management arrangements for older people. | PR |
|  | Kendall, Matosevic, Forder, Knappm Hardy and Ware [18] | 2003 | Descriptive | Home care managers in 11 local authorities | Analyse the motivations of social home care providers in England | PR, OS |
|  | Fleming, Taylor [66] | 2006 | Cross-sectional | 45 home care workers from a health and social services trust in Northern Ireland. | To explore retention of home care workers from their own perspective. | OS |
|  | Taylor, Donnelly [67] | 2006 | Cross-sectional | 99 staff members from social work, community nursing, occupational therapy and home care management in N. Ireland. | To explore perspectives on planning long term care for elderly people. | PR, OS |
|  | Venables, Reilly, Challis et al. [68] | 2006 | Cross-sectional | Services from each of the 22 local authority areas in the North West of England. | To describe the standards of home care services for people with dementia and investigate the differences in quality standards between specialist and generic home care services. | PR |
|  | Sandhu, Bebbington, Netten [69] | 2006 | Cross-sectional | 214 respondents from the PSSRU User Experience Survey in the North West of England | Examining the relationship between individual characteristics in judgement of the service quality. | PR, CI |
| **Country** | **Author(s)** | **Year** | **Study design** | **Study population** | **Study focus** | **Home care domain*** |
| **UK cont.** | Netten, Jones, Sandhu [70] | 2007 | Cross-sectional | Users from 121 home care providers within 27 local authorities in England. | To investigate provider and care workforce influences on quality of home care services | OS |
|  | Scourfield [19] | 2007 | Descriptive | Only limited to England and Wales | Examining the effects of integration of home care services | PR, OS |
|  | Pilkington [20] | 2008 | Descriptive | Not restricted | Describing the main elements of home care re-ablement services | OS |
|  | McLeod, Baywaters, Tanner, Hirsch [21] | 2008 | Descriptive | Voluntary sector hospital aftercare social rehabilitation in 5 UK localities. | To investigate the social care people need after hospital discharge. | PR, OS |
|  | McFarlane, Mclean [71] | 2003 | Cross-sectional | 1572 social care workers in 11 local authorities in Northern Ireland, England and Scotland | To describe workforce of providing social care and their interest in attaining education. | OS, PR |
| **Multiple country studies** | Kodner [22] | 2003 | Descriptive | Only restricted to US, Austria, Germany and the Netherlands | Describe the designs, developments and outcomes of consumer directed policies in Austria, Germany, the Netherlands and US. | PR |
|  | Carpenter, Gambassi, Topinkova et al. [72] | 2004 | Cross-sectional | 4,500 people 65 years and older already receiving home care services within urban areas selected in 11 European countries. | To compare outcomes of different models of community care in Europe. | OS |
|  | Le Bihan, Martin [23] | 2006 | Descriptive | Only restricted to Germany, Spain, Italy, France, the UK and Sweden. But research actually only takes into account Barcelona, Rome, Münster, Stockholm, Ille-et-Vilaine and a UK-site. | To make a cross-country comparison of the rights of care recipients. | PR, OS |
|  | Timonen, Convery, Cahill [24] | 2006 | Descriptive | Only restricted to Ireland, England, Finland and the Netherlands. | To describe the rationale behind introducing cash-for-care programmes and the effect on care regimes. | FI |
|  | Bos, Frijters, Wagner et al. [10] | 2007 | Cross-sectional | 4,007 clients of home care organisations in urban areas in 11 countries, aged 65+ years. | To study variations in quality of home care between sites across Europe. | OS |
|  | Onder, Liperoti, Soldato et al. [76] | 2007 | Prospective cohort | 3,292 older adults receiving home care in 11 European countries. | To explore the relationship between a case management approach and the risk of institutionalization in a large European population of frail, old people in home care. | OS, CI |
|  | Bolin, Lindgren, Lundborg [73] | 2008 | Cross-sectional | Approximately 22,000 respondents over the age of 50 from 10 European countries. | To explore whether formal and informal care are substitutes or complements and whether this differs between European countries. | CI |
|  | Bode [25] | 2006 | Descriptive | Only restricted to England, Germany and France. | Comparing elderly persons care networks in a competitive context across countries, focussing on domiciliary care. | PR, FI, OS |

* PR= policy & regulation; FI= financing; OS=organisation and service delivery; CI= clients & informal carers
