# Supplementary material for: Home care in Europe: a systematic literature review
Source: BMC Health Serv Res. 2011 Aug 30;11:207. doi: 10.1186/1472-6963-11-207 (PMC3170599; doi:10.1186/1472-6963-11-207)
Supplement: Additional file 3 — Description of home care by country and key domains. Description of home care by country and key domain, Word, Description of home care by country and key domains, Home care is described by country and the four domains, i.e. 'policy & regulation'; 'financing'; 'organisation and service delivery'; and 'clients & informal carers' per country. [file 1472-6963-11-207-S3.DOC]

**Additional file 3 - Description of home care by country and key domains**

| Country | **Policy & Regulation** | **Financing** | **Organisation & Service delivery** | **Clients & Informal care givers** |
| --- | --- | --- | --- | --- |
| **Austria** |  | In Austria, there were non means-tested care cash benefits for permanently disabled who required more than 50 hours of care. Most paid an informal carer with these benefits [22]. |  | Most paid an informal carer with care cash benefits [22]. |
| **Belgium** | In Belgium, the organisation and financing of home care is shared between two levels: the national level and the levels of the federal ‘communities’ (Flanders, Wallonia and Brussels). In Flanders, criteria for the eligibility for home care services are practically uniform. In 2003, throughout Flanders, even in rural areas, all types of services were available [29]. In Flanders, all interventions performed by nurses for patients at home are specified in the nomenclature of home nursing activities; including those jointly performed with other workers by the Federal Institute for the Insurance of Illness and Invalidity [30]. | In Belgium, financing home care is regulated at two levels: the national level and the levels of the federal ‘communities’ (Flanders, Wallonia and Brussels) [29]. Home nursing care is part of the social security system and is financed by the Federal Institute for the Insurance of Illness and Invalidity. Insurance for home nursing services is obligatory. An inadequacy of the funding system is that nursing agencies are paid for technical interventions, but not for the (psycho)social care they provide. The mode of reimbursement is related to the dependency level of patients. Those with low physical dependency need a prescription from a physician to get services reimbursed. Nursing services to patients with moderate to high physical dependency are reimbursed with a daily lump sum [30]. | Most frequent interventions in home nursing are self-care support, mobility support and (psycho)social interventions, combined with more technical interventions (medication administration, gastro-intestinal care, breathing management, circulation care, urinary care) [30]. In 2003, home care services were delivered by competing private agencies. All services are available throughout the region [29].  To make a diagnosis, nurses and home care workers use mainly personal observation, communication with relatives of the older person and they consult a GP. Colleagues and heads of departments were not involved. In two thirds of the cases, the assessment for dementia was tested without the existing formal assessment instrument. The diagnosis is the responsibility of a physician or social worker [31].  Nurses are mainly medically oriented and home care workers mainly provide home care tasks, e.g. cooking, cleaning, shopping. There are some home nursing organisations that employ nurses specialised in dementia care. In case no specialised nurses are available, nurses and home care workers provide help with daily tasks [31] .  The knowledge of dementia symptoms of home care professionals is insufficient; also, their strategies to find out the diagnosis are limited [31]. | Depressive moods are predictors of home care services utilisation by persons over the age of 65, however, cognitive impairment does not predict this use. This suggests unmet care needs among the cognitively impaired. There is a strong negative association between utilisation of services by persons over the age of 65 and co-residence. So, as people are increasingly living alone, the need for home help, cleaning, meals-on-wheels and home nursing is increasing as well [29]. The main counselling activity of both home care workers and nurses with regard to informal carers who look after persons with dementia was to emotionally support the family care givers, rather than to advise them. Additionally, nurses, rather than home care workers, advise them on how to interact with the person with dementia and inform care givers about available services. Both professionals hardly inform informal carers about dementia and its symptoms [31]. Dementia information centres have been set up on regional level [31].  In case of recipients with dementia, the use of home services by family carers seemed to be dependent on social and psychological characteristics of family carers rather than on functional and behavioural problems of care recipients.  Family carers, living with the relative, who had a more positive attitude towards home service and who used problem-solving as a coping |
| Country | **Policy & Regulation** | **Financing** | **Organisation & Service delivery** | **Clients & Informal care givers** |
| **Belgium cont.** |  |  | Home care workers are supervised monthly by social workers. Home nurses are trained to perform their work autonomously [31]. | mechanism seemed to received more services (the diversity) for their dependents. The number of contacts of the home service was negatively associated with family carers who did not live with the relative, who used avoidance as a coping mechanism and who had a lower burden of behavioural problems. Co-habiting with the relative increased diversity of services but decreased visits. This study suggested that family carers who are living with relatives with dementia are a better substitute for professional ADL care than are non-cohabitating family carers, i.e. those professional care activities that have an impact on the volume of professional care [32].  A study on family carers of persons with dementia showed that almost all family carers knew at least one home service. The average number of services received at home (home nursing, home care services, day care service, home cleaning, attending service, social work from health insurance agency, integrated home care service, meals on wheels, short stay respite care, volunteers, physiotherapist and dementia information centre) by family care givers who looked after persons with dementia and who received home services, was 2.3 and the mean frequency of visits was 30 in 4 weeks [32]. |
| **Czech Republic** |  |  | The Czech Republic was in the top three of European countries with most unwanted outcomes of home care, studied by the ADHOC study [10]. |  |

| Country | **Policy & Regulation** | **Financing** | **Organisation & Service delivery** | **Clients & Informal care givers** |
| --- | --- | --- | --- | --- |
| **Denmark** | Home care is part of the health care system. In 2001, Denmark had a national health system, but the responsibility regarding home care is split between the Ministry of Health (responsible for supervision and coordination through for instance regulation and guidelines) and municipalities (responsible for home care specifically) [33]. Policy and regulations on elderly care are set by the parliament and the central government [15]. The provision of health and social services are decentralised to counties and municipalities [15]. In 2001, about 75% of the municipalities had integrated care systems, that is, home care and nursing home care/ assisted living arrangements were organisationally integrated [33].  All citizens are entitled to health and social care. Eligibility is based on needs assessment with individuals and his or her household [15]. However, the costs and services provided differ significantly across municipalities in 2001[33]. To make service provision more transparent, municipalities are obliged to have laid down what services are provided, and are obliged to set up complaint boards [33].  Since 2003, recipients of home help may hire a private provider for assistance which is then funded by the municipality [15]. Municipalities are legally obliged to perform preventive home visits to citizens over the age of 75 [34]. This is laid down in the Home Care Prevention Act [33]. | Health and social services for care of older people are largely financed from general taxes, and largely available without user charge [15]. | In Europe, Denmark has a leading position in home care, providing nearly twice as many hours of home help for the elderly as Sweden, which holds the second position [15]. Many municipalities offer home help services and nursing-home care in an integrated way and report this as home help [27]. However, a polarisation between groups of care recipients can be identified. A limited number of recipients, usually residing in assisted-living dwellings, is receiving more comprehensive care packages. More limited packages of care are delivered to those living at home [27]. Preventative home visits are meant to increase well-being and to facilitate that the older persons make better use of own resources and sustain their functional ability for as long as possible. During these visits advice and guidance is given about possible support[34]. | Only 60% of people above 75 are receiving the obligatory preventive home visit. Determinants for preventive visits differed for men and women. Age, psychological characteristics and lack of mobility had a positive influence in the case of men, but the latter two had no or even an opposite influence for women. Social contacts of women were positively influencing the preventive visits [34].  A low sense of coherence was associated with being more likely to receive a maximum of 4 visits, instead of more [34]. |
| Country | **Policy & Regulation** | **Financing** | **Organisation & Service delivery** | **Clients & Informal care givers** |
| **France** | Entitlement to public financing of home care is based on means-testing [35]. In France, there is still a strong public responsibility with regard to home care, and hence, there are relatively uniform standards and state control. However, home nursing is more regulated than personal care (usually unskilled individual providers). Particularistic ‘local’ (clientele focused) agreements are being made resulting in disparities across the country [25].  The voluntary sector providing personal care and the public authority are involved in cooperative networks, for instance creating consensus decisions on training policies and salaries [25]. However, in several counties there is a move from horizontal cooperation with other providers and cooperation within the care system towards investing more energy in competition and cooperation with insurance companies and banks [25]. Competition has been increased through the possibility for counties to opt out of prices set at national level [25]. | Formal care for the elderly is funded through social security at  national level and through co-payments (depending on the financial situation of the recipient) [35]. Social security takes the form of a long-term care scheme granting an earmarked allowance [25]. Counties grant the earmarked allowance to every frail elderly person, based on needs assessment [25]. Low income recipients are not required to pay for their services, others are [23]. Benefits can also be attained in cash: ‘aide personalise à l’autonomie’ (benefits based on the level of dependency) [35][23].  Prices are administered at national level, but there are possibilities for counties and providers to opt out [25]. | Professional care for the elderly is mainly provided by non-profit private agencies [35]. The provision of home help in France is done by publicly funded, county licensed, non-profit organisations. Additionally, employment of private care workers by households has been encouraged by the government, and hence, organisations leasing private providers have grown in importance. Regional social service providers tended to opt for the private firms. However, the earmarked allowance scheme favours professional providers. Private firms are still scarce. Home nursing is provided by national health insurance funded independent or municipal nurses. Some municipalities have set up integrated service centres providing both personal care and home nursing [25].  In France, there is a personal needs assessment, performed by a team of professionals (doctors and social workers) who visit the persons to assess the ADL needs, their physical and psychological pathologies and their social and familial environment [23]. The assessment is more formalised (national scale) in France than in the UK and Sweden where there is a large discretionary power of the assessors [23]. | Over one-third of the French people over 75 years received formal home care. Formal home care is often mixed with informal care i.e. people having a spouse are more likely to receive formal home care [35].  Just like in Italy, recipients of community care in France have high physical and cognitive impairment compared to those in northern Europe [76]. |
| **Finland** | The state regulates which welfare services have to be available [37]. Municipalities have the legislative responsibility to organise health and social care [40]. They decide on the organisation and provision of these services, including informal care support [37].  Finnish policy aims to allow elderly to live at home as long as possible, if necessary with informal and formal care support [39]. Elderly care services aim to support this policy | Informal carers are eligible for an allowance (in 2006 it was 416 Euro per month on average) [38].  For municipalities the average cost for health and social care for patients at home was 742 Euro per week (including visits to social worker, GPs and hospitals). More than half of this was spent on social care services at home (home help, meals-on-wheels etc.). Home help services absorbed the largest part of home care expenditures (on average | The administration of social and health care may be either joint or separate [40]. Delivery of home health care and home help can be integrated [39].  Eligibility criteria for welfare services differ between municipalities, but are largely based on health status and coping capabilities [37]. Furthermore, the care effectively provided depends on a municipality’s resources [38].  In Finland, the needs are assessed (every six months) by a municipal care team which also informs clients about the availability of the vouchers [24]. After this assessment, those | In 2000, 11% of Finnish elderly people used public home help (this is an overestimation as it is also provided within residential units[26]); 7% home care; 14% support services; 2% informal care support [37]. In 2001, recipients of home care, in general, were likely to be female, widowed, living alone and with a low level of education. The mean age was 80 [40]. In 2003, 6.3% of people over 65 received home care services. On average, recipients of home care need help with 6 out of 15 ADLs. Clients of home care are most often patients with diabetes, senile dementia and psychiatric |
| Country | **Policy & Regulation** | **Financing** | **Organisation & Service delivery** | **Clients & Informal care givers** |
| **Finland cont. I** | [38]. Family members are not legally obliged to provide care or pay for formal care [38].  Eligibility criteria for welfare services differ between municipalities, but are largely based on health status and coping capabilities [37]. Furthermore, social and health care benefits are universal, independent of their financial situation and available informal care [38]. Between 1990 and 2000, home help had become more targeted towards the people with the highest needs and had increased in intensity. Hence, house cleaning has almost completely vanished from public provision and people with medium care needs had to resort to the private domiciliary care market [26].  In Finland, there is a cash for care arrangement. It is optional and was mainly financing private providers and self-employed individuals [24]. Informal carers cannot be paid by the cash-for-care programme[24]. The reason for introducing cash for care arrangements was to stimulate private sector provision to substitute the decreasing public provision (for private home help provision) [24]. Compared to three other countries with cash-for care programmes, services paid through the cash-for-care programme were subject to a high level of quality control [24].  In Finland, there is a municipal | 223 Euro per week compared to 67 Euro per home nursing visit) [39]. The client-co-payments are the same or lower for voucher users than for services in kind users [24]. | entitled to regular home care receive a valid service and care plan (with health status, functional ability and help needs agreed upon by the client and home care worker) [40].  In one study, no differences were found between municipalities in the costs and utilisation of home care. This suggests an equal distribution of home care across the country [39]. Another study suggested that there were differences only in the number of visits of home help and cleaning services, which were more frequent in larger cities [40].  The range of home care services consists of skilled nursing care, personal care, cleaning, meals on wheels, bathing, electronic alarm devices, transfer services etc. [39]  Home health care is provided by home nursing units, while social home care is delivered by home help services. Home helpers support people with ADL (e.g. bathing, dressing, feeding) as well as with IADL (e.g. shopping, cooking, domestic work) [38].  Private home nursing and home help services are used infrequently [39].  Over the years, home help services have come to provide care in residential units (or service housing). This has meant that there was less capacity to provide home help to peoples’ own home [26]. | disorders, or those needing post-operative or terminal care.[37]: there was a need to take care of people who were ill and to alleviate pain. Recipients were mostly older women, living alone with many co-morbidities [39].  Among predictors of mortality among the very old (90+) in Tampere were in 1999: not doing one’s shopping, not reading newspapers and regular need of help (which also predicted institutionalization) [36]. Most of those above 70 receiving formal care also receive informal care or voluntary help. Receiving formal care was associated with increased age, cognitive and functional disabilities (IADL and PADL).  Universality of benefits, i.e. being independent of informal care, is not always realised. In situations where children provide informal care to 70+ or 99+ parents it is more likely that formal care is provided as well, compared to situations where a spouse provides informal care. Children may act as agents seeking formal help [38]. Since men are less likely to live alone, they are less likely to receive home care [38].  Most clients of home care felt their home was the best place to live (82%). Received home care services ranked on frequency are: home help (7.5 visits per week), meals on wheels; home nursing (1.7 visits per week). One-third of home care recipients had both home help and home nursing [39].  A study showed that there were differences in the perceived need between home care workers and home care recipients: recipients assessed their own need for help to be lower [40]. |
| \ Country | **Policy & Regulation** | **Financing** | **Organisation & Service delivery** | **Clients & Informal care givers** |
| **Finland cont. II** | responsibility for quality of home care services, also for those provided by private providers. Furthermore, complaints procedures have been laid down [24]. |  |  |  |
| **Germany** | In Germany, cooperation between providers and co-governance has been slightly weakened by introduction of competitive elements. However, new non-profit alliances have been created through this and meetings are being held between providers and public organisations on national and local level [25]. | In Germany, home care is financed through the long-term care insurance[25] (administrated by subunits of the quasi-public health funds) [22], by mandatory, income-related payroll tax. In-kind benefits may be replaced with cash benefits [25] [23]. In Germany, the amount of cash benefits is dependent upon the level of disability. Recipients can choose between in-kind benefits and cash benefits (50% of the costs of in-kind benefits in 2003) or a combination [22]. All are non-means-tested and regardless of age [22]. | In 2006, in Germany there were several types of agencies providing home care: non-profit, for-profit and religion-based organisations [25]. They are competing for the same clients. The work of home care workers has become less professionalised due to a downward pressure on prices and hence time saving has become important [25]. In Germany, care centres and later municipal advice centres and the administrative bodies of the insurance (performing the needs assessment) have the role of informing clients on the available services. However, there is no central gatekeeper who could inform clients on all available services. Case management is hence perceived to be weakly developed [25].  In Germany, there is a personal needs assessment, performed by a team of professionals (doctors and social workers) who visit the persons to assess the ADL needs, their physical and psychological pathologies and their social and familial environment [23]. Germany was in the top three with the most unwanted outcomes of home care of European countries studied by the ADHOC study[10]. | It was found that clients had (experienced) more choice through the cash programmes [22]. However, it is said that there are insufficient mechanisms to support those eligible in making a choice [22]. |
| **Ireland** | Domiciliary care services are seen as the solution for the challenges caused by ageing. However, there is still a lack of community care which has led to overuse of hospitals. In the earliest policy documents on care for the aged, the government stated to prefer home care both as a means for better well-being of older people and for efficiency reasons.  The system of domiciliary care is | Home care is partly financed through taxation [24]. Care workers in the private sector have lower levels of remuneration (around minimum wage pay per hour) than their colleagues in the public and non-profit sectors. However, rates of pay are variable between providers [41]. The introduction of home care packages was supposed to complement the existing | Important recent changes in the delivery of domiciliary care of older persons are the emergence of private home care companies, cash-for-care as a policy instrument and the professionalisation of the non-profit (formerly voluntary) sector providers [41]. Currently, providers are mainly non-profit organisations subsidised by the government. Although there may still be a religious affiliation, the religious aspect has less influence than in the past. Under the pressure of private organisations | Informal carers are said to be taken for granted. In 2006, the carer allowance that was available was means-tested [11]. |
| Country | **Policy & Regulation** | **Financing** | **Organisation & Service delivery** | **Clients & Informal care givers** |
| **Ireland cont.** | fragmented. There are many different ways in which delivery is organised and how home care is assigned (in some areas a means-test is required, in others just a medical card and in some co-payments are required while in others they are just encouraged) [11].  Home care was originally only aimed at the poorest. This has changed now (all persons over 70 years of age are in principle eligible to this service) [11].  There is a lack of regulation of the private and non-profit domiciliary care sectors. This facilitates more flexibility with regard to qualifications, training and monitoring of the quality of work. Care workers in the private sector have weaker social rights than their colleagues in the public and non-profit sectors [12,41].  Recently, home care packages were introduced at the national level [12]. This is one of the recent major shifts in the home care sector creating an increase of private care provision. These shifts have resulted in a need for regulation on home care quality, monitoring and coordination. In these aspects the home care sector lags behind that of institutional care. Regulation would require extra costs, while private home care provision has been encouraged for reasons of cost reduction [11]. The reason for introducing cash for care arrangements in Ireland was mainly reducing institutional care and through this costs [24]. In Ireland these ‘home care grants’ were optional, could not be used to pay | provision of home care services by non-profit and public providers. The importance of this ‘financing’ mechanism is growing and, in some areas, is even replacing the benefits in kind [12]. Payment of providers differs. Non-profit providers are paid based on the number of hours provided in the past. For-profit organisations are mainly paid on the basis of the number of home care packages they are contracted for [12]. | non-profit organisations have been more professionalised [11]. The cash-for-care scheme and new trends of competitive tendering (for receiving a contract to provide home care) have led to the introduction of competition in the greater Dublin region [12].  Partly as a result of the cash-for-care regulation, there is currently a mix of state-funded non-profit, for-profit and public provision [12]. Non-profit sector organisations recruit care workers almost exclusively from their immediate surrounding areas, while the private agencies tend to draw on a larger and more disparate geographical area both for their workers and for their clients [41]. Whether training is being paid for by employers also depends on the type of provider: in the public sector training is usually paid by the employer, while the employee is expected to pay for its own training when working in private agencies [41].  The composition of the care workforce is more diverse (e.g. in nationality of workers) in the private sector than in the public and non-profit sectors. The sectors also widely differ in terms of qualifications, training requirements and security checks. Private sector care workers have more flexibility than their public/non-profit sector counterparts in terms of the range and combination of care duties. Public sector workers (‘public health care assistant’) carry out personal care duties exclusively whereas most non-profit sector workers are focused on domestic work, and carers in the private sector combine personal and domestic care [41].  The health care assistants provide intensive personal care and non-medical care to people at home [11].  A trend and concern in Ireland is the growing number of home care agencies acting as intermediaries: they provide people in need with self-employed home carers (and therefore |  |
| Country | **Policy & Regulation** | **Financing** | **Organisation & Service delivery** | **Clients & Informal care givers** |
| **Ireland cont. 2** | informal carers and was used for private providers of home care and self-employed individuals [24]. The freedom of spending the Irish ‘home care grants’ differs across health authorities. In some areas recipients pay and employ care providers themselves, in others they do not and the care providers are paid directly only after the services are provided [24].  Some minimum requirements exist for private and non-profit providers to be contracted by the HSE [12]. |  | difficult to control) [12]. In addition to these agencies, the for-profit sector consists of companies (placing, directly employing and supervising carers). Carers employed by intermediaries generally have more precarious work contracts [41].  The grey sector is also important in the home care sector in Ireland [12].  Even tough persons over 70 are in principle eligible to home care, due to a lack of resources the services available may be very little (e.g. max. 2 hours a day) and often co-payments are required. Hence, privately financed care is increasing [11]. |  |
| **Italy** | Eligibility for home care is associated with functional impairment and co-morbidity [42]. | Allocated services depend on the available resources (not completely related to the level of needs) [23]. In Rome, there are furthermore even income thresholds for receiving home care [23]. Cash benefits to pay for care services were available in Rome [23]. | In Italy, very little formal care was available, compared to other countries in Europe [72]. Receiving home care was even dependent upon the available resources [23]. Italy was in the top three of European countries studied by the ADHOC study with most unwanted outcomes of home care [10].  In Italy and Spain the assessment may depend per region and assessment team. | Lack of informal support services seems to be an exclusion criterion for access to the home care programmes. This reflects the general attitude of agencies that home care assistance should be delivered to older clients with severe functional impairment but with an efficient informal support system [42]. Recipients of community care in Rome have high physical and cognitive impairment compared to home care recipients in northern Europe [76]. |
| **Netherlands** | In 2005, eligibility criteria to home care were set nationally [44].  The cash (for care) benefit, called Personal Budget, is not restricted to a certain age. The amount does depend on income [22]. The reason for introducing cash for care arrangements in the Netherlands was to increase freedom of choice and decreasing the role of traditional (inflexible) service care provision and to create a more demand oriented approach [24]. The personal budget was optional and could be used by informal carers.[24] | In 2006, home care, like other long-term care, was financed through national social insurance: the ‘Exceptional Medical Expenses Act’.[44] The optional personal budget covered about 75% of the costs of equivalent direct provision. It could also be used to pay informal carers. [24] Means-tested client-copayments exist with a maximum limit [24]. | Most of the direct care providers are established home-care organisations and non-profit agencies.[24]. In the Netherlands, both professional care with instrumental activities of daily living (domestic aid) and activities of daily living (physical and personal care), technical nursing and psycho-social care are provided at home [47]. A range of more than 120 items of home care services could be grouped into four groups: domestic help, physical care, social support and nursing. Usually services from different groups had to be delivered in combination [81].  In the Netherlands, needs are assessed by independent assessment agencies (in 2000 85 in total) [24]. The needs assessment is | Most clients need a combination of different home care services [81]. Which mix is assigned, depends on the type of client. Chronically ill elderly people are more often referred for domestic help only. Applicants with psychosocial disorders are more often judged eligible for packages including social support. Patients discharged from hospital are more likely to receive just domestic help when they are slightly disabled, and packages including physical care when they are more disabled [81].  A 2005 study (on 2000 data) showed that the choice for a specific type of long-term care (different home care types, nursing home care or residential care) was not only decided based |
| Country | **Policy & Regulation** | **Financing** | **Organisation & Service delivery** | **Clients & Informal care givers** |
| **Netherlands cont.** | Compared to three other countries with cash-for care programmes, services paid through the Dutch programme was subject to a high level of quality control [24]. In the Netherlands, there is a special Needs Assessment Decree covering home care, but also nursing home and residential care. This also pertains to personal budgets. The decree describes several organisational aspects and prescribes that needs assessment needs to be integrative, objective and independent. Furthermore, it requires assessment agencies to lay down the types and number of carers in an assessment report. The exact ‘topics’ on which they should report are also laid down by this decree. [47]. |  | independent in that these assessment agencies are independent from providers and financing agents [47]. Assessment was found not to be dependent on service availability, but the assessment procedures were then not completely standardised yet [44]. However, in case of singular care needs, providers may still indicate care (for efficiency reasons). One quarter of home care providers in 2000 have said that they perform these assessments. Hence, assessment may not be completely independent in the Netherlands [47]. The needs are assessed integratively with needs for residential, day and nursing home care [47]. Another aim of the Needs Assessment Decree is that needs assessment is objective. The government recommended the use of a standard assessment form. Its use is quite common with the assessment agencies, but only half of the providers of mandated home care agencies (for simple indications) used them. They create objectivity, however, they were said to be impracticable; home care agencies said that the forms needed to be complemented [47].  The Dutch government is trying to increase standardisation of the procedures [44].  At the intake and during subsequent needs assessment, information is gathered about the applicant’s living situation, health status, impairments, disabilities, use of other health care services and available informal care. Health problems are categorized in an ICIDH-based classification system, which  provides the formal basis for the recommendations made to the indication committee regarding the required home care services. In addition, the reasons for | on disease and disability level, but also age, household composition, housing and use of other health services. This study also showed that about 6% of the decisions made were negative: no care was assigned. Most long-term care assessments (also including nursing home and residential care) were for domestic help (30.0%), and, secondly, physical care plus and nursing care plus (all three are home care packages). One third of the applicants for long-term care is referred by a GP, specialist doctor or hospital. Recipients of domestic help are more likely to have IADL impairments and be female, than other types of recipients. Persons with cancer and diabetes are more likely to be assigned home nursing. Men are more likely to be assigned physical care and home nursing, than women[44].  Overall, half of the independently living older people studied prefer formal care to informal care. Restricted to personal care, even 80% prefer formal. Care preferences are influenced by individual, social and experiential indicators, and attitudes towards care.  Positively modifying the public opinion on informal support may both favour preferences for informal support and increase the general willingness to provide informal care [43].  A study among informal carers showed that one third of them received respite care services; on average 18 hours per week. Users of respite care were generally satisfied with it [46]. |
| Country | **Policy & Regulation** | **Financing** | **Organisation & Service delivery** | **Clients & Informal care givers** |
| **Netherlands cont. 2** |  |  | application and the current use of health services play an important role in the decision to allocate services [81]. Usually, the assessor visits the patient at home [47]. After needs assessment, the health insurer has to confirm the care entitlement [47] and then the client can opt for either direct care provision or a personal budget [24]. According to health insurers the switch from providers performing assessments towards independent assessors has led to a more independent and comprehensive assessment, but not necessarily to a more objective assessment [47].  Burnout, which is not uncommon among home care workers, is strongly related to job demands, in particular emotional demands and harassment from patients. Autonomy, social support, and opportunities for professional development appeared to be crucial buffers against burnout. Job resources can compensate for negative effects of job demands on burnout [45].  The main problem related to the Personal Budget was coordination of care as in 2006 many budget recipients hired several workers to decrease employers’ responsibilities [24]. |  |
| **Norway** | LTC is a responsibility of the municipalities [49]. Control of the quality of services belongs to the responsibility of municipalities.  Quality control of LTC services is a governmental priority, reflected in stimulation of internal measures to comply to laws and regulation[49]. However, no regional or national register exists of clinical outcomes of LTC [49].  From 1994 onward, personal assistance has become an option and would be formalised by law in 2000. | LTC is publicly financed [49]. | Provision of LTC is under the responsibility of municipalities. Within municipalities the responsibility is delegated to nurse leaders; they are also responsible for the quality of services [49].  In municipalities, the provision by care teams was separated from the municipal purchaser unit responsible for assessment and purchasing. The latter would inform and assess clients independently and would increase efficiency and would increase transparency of home care providers’ work. More paperwork was hence introduced. [13] The general focus of quality improvement is on general aspects rather than on technical aspects. Safe use of medication has been a priority. In about two- | In 1998, there were 300 users of personal assistance (granted by 192 municipalities). In 1996, 75% of the users of personal assistance were below the age of 50 and better educated than the average disabled person, but less likely to be employed. On average, they received 34 hours per week in 1996. The big and average consumers of personal assistance (in terms of hours) mainly receive traditional services as a complement, while in case of small consumers personal assistance supplements traditional home care.  The introduction of personal assistance has decreased the dependency on informal care for every group of intensity of use (small, average |
| Country | **Policy & Regulation** | **Financing** | **Organisation & Service delivery** | **Clients & Informal care givers** |
| **Norway cont.** | It was realised through efforts of organisations of disabled persons. Personal assistance in Norway is primarily (but not exclusively) aimed at physically disabled persons with comprehensive needs. Recipients appoint the assistants and decide on the services and time at which they should be provided. In 1999, most personal assistants were employed by the municipality (others by clients or associations of disabled persons). Personal assistance was meant to replace normal service provision partially. The Ministry recommended a combination of services [48].  The consumerist approach in Norway has been linked to the separation between the purchaser provider role, the introduction of voucher arrangements and competitive tendering. There is a friction between (too) high expenditures and high expectations by the population about the government regarding care for elderly persons. Consumerism, and instruments introduced based on it, also aimed to increase efficiency (with little effect on the number of recipients). Management tools such as quality management, user surveys and contractual arrangements were introduced. A conflict seems to exist between the contractual arrangements (assessors stipulate what is to be provided) aimed at creating more transparency for the sake of recipients on the one hand, and decreasing flexibility for the sake of the client [13]. |  | thirds of units user surveys were used to improve quality of care. Larger municipalities usually have a more comprehensive programme for quality improvement [49].  In order to improve care, integrated service teams were set up at municipal level [13]. | and big). Reasons for the client’s application for personal assistance were to have a more flexible service, self-determination, fewer professionals providing care, to choose their own provider and be able to live a more active life. In general, users were satisfied with personal assistance. The small consumers were the least satisfied [48]. |
| Country | **Policy & Regulation** | **Financing** | **Organisation & Service delivery** | **Clients & Informal care givers** |
| **Poland** | In 1995, the profession of family nurse was introduced. Educational levels are set. Family nurses follow training for nursing and thereafter they follow additional training. The tasks they perform are laid down in a Ministerial Decree. Their tasks are health promotion, preventative health care, some diagnostic tasks and nursing, medical and rehabilitative services [8]. | - | In Poland, home nursing visits are performed by doctors, by family nurses employed by doctors and contracted by the National Health Fund (for patients registered with that family doctor), and, since 1998, by self-employed family nurses contracted by the National Health Fund (contracted for specific tasks). Finally, the more rare option is that nurses can be employed by a public health centre. The possibility to practice independently has led to an increase of home visits being made. But between 2003 and 2006, the number of home visits by family nurses declined (since 2005 home visits are only made during the day and week).  Most common activity of family nurses in a small Poland town were interventions, i.e. procedures requested by family doctors such injection and decubitus. Health education was the second most common reason for home visits and a third most common reason was performing diagnostic services. Between 2002 and 2006 their health promotion decreased, while interventions and nursing tasks (i.e. interviews on living conditions, diagnostic tasks without doctors orders) increased [8]. | In 2006, about 23.9% of respondents to a survey in a municipality with one of the lowest population densities in Poland reported having received a home visit. The largest group of recipients were those over the age of 75 (this group was 7.3 times more likely to receive a home visit than a person between the age of 15-64) [8]. |
| **Portugal** | Integrated care has recently become an issue in Portugal. However, already in 1997 the Programme of Integrated Support to the Elderly was laid down. This aimed to maintain the autonomy of the elderly in their own homes, providing support to family members and to promote training of providers of care [50]. Still, care and health providers have been separated due to different rules and jurisdictions apply to them and distinct budgets [50]. | The National Health Service, is a universally accessible service for protection of health, and is financed through general taxes. Expenditures usually exceed the budget limits. Additionally, 10% of the population is covered by voluntary insurance and 7% by mutual funds [50]. | Social community services are said to be insufficient. The main providers of social services are private non-profit providers for social solidarity (IPSS). In some areas, partnerships with municipalities, non-profit providers and regional health administrations have been set up [50].  Care and health providers have been separated due to different rules and jurisdictions that apply to them, and distinct budgets, different cultures and approaches to care. Related problems that have arisen are the existence of multiple entry points and deficient transmission of information between institutions and professionals. The Domiciliary Support Service (SAD) (providing nursing, | - |
| Country | **Policy & Regulation** | **Financing** | **Organisation & Service delivery** | **Clients & Informal care givers** |
| **Portugal cont.** |  |  | personal care transportation and meals) was expected to have a more integrated approach. It is aimed at satisfying the basic needs of clients, providing physical and social support (also to the family) and collaboration with health care providers. A study in Aveiro showed that in practice they do not provide more integrated care. Especially the integration with health centres is rare. Here, client records were not exchanged with other institutions. Furthermore, their services were mainly social services. Although physiotherapy (8%) and nursing (35%) was provided by the SADs, these services were evaluated by clients as being bad or very bad [50]. |  |
| **Slovenia** |  | For most elderly persons home health assistance is expensive, especially for daily care [9]. | Home health care assistance is developing, but usually still available only in urban areas. Additional to home health care assistance those in need can be visited by a nurse. However, these visits can not be made frequently [9]. | - |
| **Spain** | Provision of health care services in Spain has been decentralised to the regional governments. The Personal Community Care Service System is run by regional governments as well as municipalities. Eligibility depends on needs and income [51].  In 1993, a National Gerontologic Plan was issued. In 1999, Madrid developed a Regional Plan for the Elderly [51]. | The health care system and Personal Community Care Service System are publicly financed. The latter is financed by local and regional governments. But these resources are low and coverage is very low [51]. In Barcelona (not nationally decided) there are even income thresholds for receiving home care.[23]. Cash benefits to pay for care services are available in Barcelona [23]. | The main source of help for dependent elderly persons is their family [51]. Formal community care services are scarcely available. They are provided by the public as well as the private sector [51].  In Spain there is a personal needs assessment, performed by a team of professionals (doctors and social workers) who visit the persons to assess the ADL needs, their physical and psychological pathologies and their social and familial environment [23].  In Spain, the assessment may vary per region and assessment team. Allocated services depend on the available resources (not completely related to the level of needs). In Barcelona, an increase in dependency is not always associated with an increase in service provision [23]. | Families provided almost all assistance; only few reported to receive additional formal services [51].  Unmet home care needs among community dwelling elderly persons were associated with low income, lower education, living alone and symptoms of depression [51]. |
| **Sweden** | Since the early 1990s, the Swedish government has sought to substitute | Home help in Sweden is a comprehensive and needs-assessed | About 7.6% of the elderly persons receive Home home Helphelp, and about the same proportion is in institutional care [59]. Between | A 2006 study showed that in 2003, 54% of the Swedish elderly persons who were frail and lived alone used public home help services. |
| Country | **Policy & Regulation** | **Financing** | **Organisation & Service delivery** | **Clients & Informal care givers** |
| **Sweden cont.** | institutional care for home care [62]. Services to people at home can be provided through two programmes: Primary Health Care (usually organised by county councils) and Home Help (run by municipalities) [55]. In addition to home assistance, social services of municipalities include institutional care and long-term health care for the elderly persons [53].  One study identified three levels of structural integration of social and health care provided at home. These levels were related to the responsibility for and the provision of ‘home care’ (referring to home health care). About half the participating municipalities provided and were responsible for ‘home care’ as well as home help. In 26% of the municipalities home care was the responsibility of the county and delivery of qualified health care was done by the county’s district nurses, but less qualified health care was delivered by the municipality’s assistant nurses. In 23% of the sampled municipalities, ‘home care’ was provided by and the responsibility of the counties. In a majority of the county councils (55%), either all municipalities in one county took over all responsibilities or no municipality took over the responsibility [61].  Rights to and application for home care are regulated by the Social Welfare Act allocated on the basis of need [58]. Eligibility to home help, described in the Social Service Act, is based on the need for help and on | public facility providing domestic services and personal care. Clients pay an income-related fee for each hour of help [14] and a large part is financed by municipal taxation [57].  Amounts of client co-payment may vary between municipalities [80]. The co-payments also depend on the costs of care packages that are required [23].  Cash benefits to pay for care services are available in Sweden [23]. The way the amount of cash benefits are decided on is part of the overall needs assessment [23].  The expenditures on personal assistance (encompassing more than home nursing, personal care and domestic aid) have increased between 1994 and 2004. Personal assistance provided in a person’s own home is free of charge for persons eligible. The costs are then shared between the state and the municipality in case of personal assistance or a personal assistance allowance [16]. | 2000 and 2004 bed supply in care institutions decreased by 11% and home care increased by 9% [80]. The Home help system provides domestic aid and personal care (help to go to bed, or get to the bathroom). The public home help service usually only provides infrequent domestic aid and cleaning of just one room [60]. Home care in the evening or night usually concerns personal care or help with medication [55]. In many municipalities assessment and provision are organisationally split: the home help officer involved in assessment does not provide the care. He/she does have some financial responsibility: they should prevent overspending. The needs assessment is performed by the social welfare committee’s care manager [57]. The assessment is performed mainly at the patient’s home or in hospital before discharge [28], to assess the ADL needs, their physical and psychological pathologies and their social and familial environment [23]. The needs assessment process is a problem-solving event in which the applicant’s accounts of care needs are negotiated and responsibilities and contributions of family and the public system are balanced [58]. Care managers decide on the intervals and frequency of care based on the loss of autonomy, the type of care required and number of hours needed, but currently procedures are based on international standards and not only left to the experience of care managers [23]. In Sweden, care is assigned only for a few months. In this way care can be monitored regularly [23]. Inadequacy of needs assessment was mentioned as a problem as many health personnel under-report complaints. Needs assessment mainly focuses on aspects of physical ability (rather than on social and psychological aspects) [80].  There are variations in the type and the extent | The average home care client uses 32 hours per month, but the variation is large. Very few use more than 200 hours per month [14].  Of the elderly persons in need of help with PADL, 65% received public home help. Informal care is received by many more. Little variations were found across municipalities and thus uniformity in needs assessment seems to exist [60]. There is no association between local variation in home help coverage and unmet needs among older people [52].  The 2006 study showed a decline in the provision but also the need for home help between 1988/89 and 2002/03; and that there seems to be a tendency for a shared responsibility for care between the public services and the family instead of full public responsibility [60,62]. The proportion of elderly persons receiving home help has decreased, while the proportion receiving informal care increased over last decades [62,80]. This suggests that informal care substitutes formal care [80]. 1994-1996 Data showed that informal care seemed to suppress the use of formal care services [56]. Still, two-thirds of people with municipal home care relied on informal care as well [62]. Hence, possibly, there has been a shift from formal care as a substitution for informal care to a complementary role [62]. Offspring’s increased support does not support the common belief that employment and other commitments hinder women providing informal care [52].  Generally, the same factors predict both the receipt of home help and institutionalisation.  Predictors among those living alone are: dementia, functional limitations and depressive symptoms. Predictors among demented cohabiting people are: depressive symptoms and dependence in ADL. Higher level of education increases the likelihood of institutionalization [75]. Another study showed |
| Country | **Policy & Regulation** | **Financing** | **Organisation & Service delivery** | **Clients & Informal care givers** |
| **Sweden cont. II** | whether needs can be met in other ways (only the spouse is obliged to provide care) [60].  Caregivers can be eligible for support by the municipality (for instance, payment for care, relief services and day care) in accordance with the 1998 Social Service Act [53]. The 1997 Elderly Bill stipulated the need to develop services for caregivers. The home help system is seen as an indirect source of support for informal caregivers [53].  Despite cuts in the 1980s and 1990s, Sweden’s home help system is relatively comprehensive and universal [14]. There has also been no official policy change related to these cuts [57]. Over the past 15 years, formal support to older people with lower dependency has been decreasing and need for public social care support has been redefined [80].  It has been reported, however, that this has not resulted in more unmet needs, because of higher efficiency and effective targeting [79]. Further cut-backs in public old-age support are expected to be compensated by more informal care [53].  Children are not formally obliged to take care of their frail elderly parents [62]. Relieving families of the burden of care for the dependent family members has been an explicit policy goal in Sweden [60].  There is however a debate going on about the role of family and state in the care of elderly persons. Between 1994 and 2000, public home help for the elderly in the community is decreasing more than institutional |  | of home help services, but clear evidence for the existence of inequality is absent [79]. A study in a Stockholm district showed that need was still the main and most important predictor. However, older persons and persons living alone were assigned significantly more hours of home help [57]. At national level, higher coverage rates for home help needs were found in municipalities with a population being older and with more single elderly persons [79]. Regarding the needs, home help services have become more targeted towards those with the highest needs and shifted from IADL to PADL and ADL[79].  During the past decades a decrease in formal care has been compensated by an increase in family care (reverse substitution). Frail elderly persons are increasingly offered other services, such as transport, meals-on-wheels and security alarms. These serve many more than the clients of formal home help [14].  Attempts are being made to better integrate formal and informal care and thus to foster synergy between both. New legislation aims to improve direct support to carers [59].  Over time, home help coverage and service intensity varied inside municipalities, related to the supply of home help workers and level of expenditures. In the municipalities with higher median incomes and higher unemployment rates and relatively higher expenditures, the services were more intensively delivered. Furthermore, the effects of the ratio of women compared to men on the service intensity also changed over time [79].  There were around 50,000 personal assistants in 2003. Those entitled to a personal assistance allowance can choose their provider. Most choose their municipality. Other possible providers are cooperatives or companies. There is also the possibility for clients to employ an | that those with higher education were more likely to receive public home care [56]. Compared to patients living in special accommodations, those receiving formal care at home are younger, more likely co-habiting, more often male and more likely to have less physical disabilities and report heath problems less often [62]. In a district of Stockholm, co-habitation and age are also predictors of the numbers of hours of home help received. Environmental (home adaptations and informal care) and structural (care manager) characteristics only marginally influenced the number of hours received. This study performed in Stockholm could just explain 51% of variation between home help recipients [57]. The odds of clients buying private care are lower when one is ADL dependent as opposed to not being ADL dependent [56].  Dissatisfied home nursing clients complain about aspects of care (e.g. pain alleviation; safety; spiritual needs; decision-making process and information given) as well as organisational matters (e.g. well-functioning of the organisation; possibility to reach staff; and always meeting the same nurse) [52]. Dissatisfied patients rated their physical health poorer than did satisfied patients [52]. Few appeals to courts have been made about total denials of home help [14].  The quality of care seems to be rated more positively by clients than by professional carers with regard to the medical-technical competence of caregivers and with regard to the physical and technical conditions. This also holds for the socio-cultural care environment. Home care users rated the interpersonal aspects of care as less important than medical technical  and physical technical aspects [54]. The Swedish Health and Medical Services Act was revised in the 90s to give care recipients more |
| Country | **Policy & Regulation** | **Financing** | **Organisation & Service delivery** | **Clients & Informal care givers** |
| **Sweden cont. III** | care. Coverage for home help services has decreased and family members and privately hired workers are increasingly involved [74]. |  | assistant themselves (in 2004 just 4%) [16]. There are no educational requirements for personal assistance providers, and many were said to have no special/formal training. The satisfaction about freedom of choice (in providers and tasks performed) depended on the type of providers (municipality or co-operative). On the whole, 90% of the personal assistance allowance recipients in 2005 were (rather or very) satisfied with personal assistance [16].  In just 10.5% of the municipalities public care was complemented by non-public care [61]. It is up to county councils and municipalities how they organise home care for the elderly: whether they leave both home care (home health care) and home help (social care) to the municipalities or whether they split the responsibility and provision of services between the two levels [61].  A study identified three levels of structural integration of social and health care provided at home. These levels were related to the responsibility for and the provision of ‘home care’ (referring to home health care). About half the participating municipalities provided and were responsible for ‘home care’ as well as home help. In 26% of the municipalities, home care was the responsibility of the county and delivery of qualified health care was done by the county (district nurses), but less qualified health care was delivered by the municipality (assistant nurses). In 23% of the sample municipalities, ‘home care’ was provided by and the responsibility of the counties. In a majority of the county councils (55%), either all municipalities in one county | influence on their treatment. In contrast, it was found that there was an imbalance of power in the needs assessment for home help/care for elderly. A reason for this is the lack of knowledge of the applicant about the needs assessment process. Assessors mainly kept to the ‘general guide of principles of help’ in their decisions showing the lack of influence of care recipients. The recipients perceived this guide to determine the services they would receive [28]. |
| Country | **Policy & Regulation** | **Financing** | **Organisation & Service delivery** | **Clients & Informal care givers** |
| **Sweden cont. IV** |  |  | took over all responsibilities or no municipality took over the responsibility. Moreover, in 85% of the councils two thirds of the municipalities had the same model of home care integration. The integration model used in municipalities was found to be independent of their number of inhabitants and of other organisational reforms taking place after the Ädel-reform: only 1% of the municipalities taking over the responsibilities of ‘home care’ had another change in home care organisation. The fact that the level of structural integration of home care and home help was so homogeneous across municipalities in one county is said to point to a certain inertia at the network level: something is stopping municipalities from taking on a different level of integration than other municipalities in the county [61].  In a county where the provision of home health care and home help were split between county and municipality it was found that there were problems with coordination in the actual care process as well as in assigning the care. One difficulty in the former self-treatment. In the case of self-treatment not home health care but home help providers would be involved. However, it was not laid down what self-treatment contained. Also, the instrument to coordinate care between professionals was insufficiently used. This instrument was a binder which contained information on home care interventions performed and used for facilitating communication between care providers [17].  The National Board of Health and Welfare obliges care providers to share information and to set up a (home) care plan meeting with all parties involved before hospital discharge of the patient [17]. The initiation to seek care can be undertaken by the person in need, their family or through hospital discharge planning, home help service or primary care doctors[28]. |  |

| Country | **Policy & Regulation** | **Financing** | **Organisation & Service delivery** | **Clients & Informal care givers** |
| --- | --- | --- | --- | --- |
| **Switzerland** | Health services are organised at the level of the country’s 26 relatively autonomous cantons, under responsibility of local public health departments [78]. | Health insurance is compulsory and organised at federal level. Funding of home health agencies stems partly from public sources at federal, cantonal, and community levels, partly from the compulsory health insurance (for nursing and basic personal care) and partly from clients private payments [77]. | Home care agencies are staffed according to standards based on the population served. They offer coordinated nursing care, basic personal care, domestic aid, occupational therapy and social services. Home health agencies are run by private, non-profit organisations [77].  Medical advisors work with the home health agencies, but medical care is provided by the GPs [77].  Demand for home health services is increasing as the length of hospital stays is decreasing [77]. | Activities of home health agencies are not limited to chronically dependent elderly persons; almost one quarter of clients are under 65 [77].  With certain conditions, hospital-at-home care is considered an alternative to hospitalization. More severely ill patients tend to prefer to be hospitalised to avoid overburdening their caregivers and to feel more secure [78].  Unscheduled services of home care agencies are predicted by the presence of a urinary catheter, incontinence and the need for assistance in bathing among clients [77]. |
| **United Kingdom** | Supporting people at home and preventing delayed hospital discharge are explicit policy aims of the UK government [67].  Already in 1989/1990, the government, laid down their aim to increase choice for service users and carers [63].  Furthermore, client-tailored care was being stimulated through asking providers to introduce suitable management systems [65]. Furthermore, they have introduced integrated care trusts in England and Wales (laid down by law).  Additionally, in England and Wales differences in user charges between the NHS and local authorities providing intermediary care were countered through the Community Care Act. However, the ‘integrated domiciliary services’ are time limited (only a certain period after hospital discharge). Ordinary care services are less integrated [19].  In the UK, the above mentioned intermediary care has become a major | Home care is partly funded through national general taxation distributed between the local authorities [24]. Additionally, in the UK means-tested co-payments based on the costs of care packages were required [23]. Cash benefits for care services were available in the UK [23]. Also informal carers could be paid in this way [24]. | With the growth of the number of providers, choice for home care users and carers has increased. This increased choice may be felt mostly by purchasers. At operational level, choice also increased as a result of a wider range of more available services.[63].  In Northern Ireland, there has been a move from a traditional domestic help service to a service that aims to meet a wide range of needs including personal and healthcare needs [66]. A shift from in-house provision by local authorities towards independent providers is taking place in England [70]. Domiciliary care in England [18] but also personal care in England and Wales [19] are mainly provided by the independent sector under the responsibility of local authorities. In England in 2006, 80% of the domiciliary care providers were private or voluntary organisations [19].  In contrast, in Northern Ireland, almost all services were provided by the in-house statutory scheme known as ‘Home Care’ in 2002, after fragmented provision during 1992-2002 [66].  Both voluntary and private providers have become increasingly influenced by new public management (target setting, benchmarks etc.) | More extensive use of statutory (by home helps, nurses, meals-on-wheels) and private home care services (privately paid) was associated with higher age, not owning a car, being widowed and worse self-reported health.  Determinants of the use of both kinds of services were: poor physical functioning, poor emotional health, problems with cognition, foot problems and falls [64].  Women with higher education use fewer statutory services [64].  A study in one metropolitan area in the North West of England showed no significant difference in user satisfaction between users of different providers of domiciliary care (personal care, domestic support, sitting services and assistance with disability) or between those living in different areas. The method and timing of services does seem to influence the user satisfaction with home care. Tea-time visits, which are usually the final visit of the home care workers, were associated with low user satisfaction, and also with not receiving care on time and with not enough time being spent at the patient’s home according to the patient. Only needing help with bathing was also associated with higher |
| Country | **Policy & Regulation** | **Financing** | **Organisation & Service delivery** | **Clients & Informal care givers** |
| **UK cont.** | strategy of the NHS plan and is discussed in the National Service Framework for Older People [21].  The ‘Direct Payment’ (DP) schemes were also introduced to increase freedom of choice and client-tailored provision and decreasing the role of traditional (inflexible) service care provision. The DP is meant to be complementary to other health and social services [24]. Municipalities are obliged to offer DPs [24]. Quality control of services paid through the cash-for-care is limited [24]. The payments do not cover all costs [24]. Several barriers to use the DP exist, e.g. a lack of knowledge on the availability of the payment, professionals wanting to keep control over funding, bureaucracy and opposition by some local authorities and public trade unions [24]. Development of a skilled workforce is seen as essential to the quality of social care. Hence, several initiatives to enhance the competence of this workforce had been set up. The Training Organisation for Personal  Social Services has been established to oversee the quality of the workforce, the General Social Care Council England (also available in other areas of the UK) to regulate the workforce and promote education and training and the National Care Standards Commission to regulate social and health care providers [71]. |  | [25]. Managers in the private sector are more likely to be better able to cope with the recent policy changes in the UK than voluntary sector managers. Their intrinsic motivation fitted the new environment. The motivations of voluntary organisations seem less adapted to the new policy environment. For example, relatively more managers in voluntary organisations have problems in negotiating contracts with Local Authorities in England than do managers in the private sector [18].  In England, needs assessment for social domiciliary services takes place through a single assessment process performed by a care manager [18]. Local authority social service departments decide upon the care plan (changed every 6 months) and case managers of social service departments inform and help clients with Direct Payments [24]. When care packages are assigned, the care provided is monitored after 6 weeks by councils with social service responsibilities. A study showed that few care packages were revised after this monitoring. In contrast, recipients of home care ‘re-ablement’ more often improved their level of dependency after these six weeks [20].  Home re-ablement, provided by adult social care services, aims to increase the self management of dependent people through learning and relearning skills. Homecare re-ablement is also part of the needs assessment process, hence the needs assessment is made over a period of time.  Home care re-ablement was implemented or was considered by them to be implemented in 130 of the 150 councils with social service responsibility in 2007 [20]. | overall user satisfaction, expectedly as users may prefer these tasks to be performed by professionals. People with a high risk of falling were on average more satisfied with the home care received. Home owners more often reported high satisfaction with receiving domiciliary care for less than 5 hours per week [69]. |
| Country | **Policy & Regulation** | **Financing** | **Organisation & Service delivery** | **Clients & Informal care givers** |
| **UK cont. II** | The inconsistency in the approach to assessment and care management across English municipalities greatly concerns the current government [65].  The needs of carers themselves have been officially prioritized in the 1999 National Strategy for Carers [68].  In England, market forces (a long tradition) and public control of quality of social care, including home care, are complemented by a voluntary sector and hence by co-governance [25]. Infrastructural issues are dealt with through Local Strategic Partnership Boards and sometimes meetings between local authority and representatives of providers [25]. Non-profit organisations are advocating more market regulation (for instance on quality and single assessment). This regulation has been partially introduced [25].  In the UK, several policies have been introduced to endorse high quality home care and standards for maintaining them. It started with the introduction of the ‘Best Value’ regime, and went on with regulations on domiciliary care agencies, the introduction of National Minimum Standards for domiciliary care setting registration requirements, and the obligation for councils with Social Service Responsibilities to measure the user experience[69]. In 2003, the National Care Standards were introduced for domiciliary care in England [18]. In the UK home care provision is monitored by National Minimum Standards for domiciliary care [68] and attention to ‘risk management’ within health and social |  | Intermediary care, a major strategy of the UK government, aims to offer a structured mix of short-term health and social care to assist recovery after a one-window assessment procedure [21]. Problems with intermediary care were too early discharge of patients, poor coordination and that unlike patients providers give low priority to domestic aid after discharge [21]. In England and Wales, problems arising at the interface of intermediary care and mainstream social care services are the unwanted change of professional, the (sudden) introduction of client co-payments, inappropriate use of respite care (due to a lack of resources) and lack of appropriate care assignment leading to overuse of intermediary care [19].  A survey in several municipalities across the UK showed that almost all social service (public) home care workers work with older people, were relatively old and 80% worked part-time [71]. In Northern Ireland, home care workers have an increasingly demanding job due to more complex health and social care needs and regulation in terms of quality and risk. They are dissatisfied about irregular working hours, lack of management support and workload pressures [66]. Home care workers in Northern Ireland face many hazards ranging across access issues, hygiene, manual handling, aggression, domestic animals and safety of home equipment [67]. In the UK, two fifths of social service departments reported problems with reaching requirements regarding home care workers. Social workers usually do not have a formal qualification or education. Social service home care workers are less likely to have followed education and training than residential care workers, and are also less interested in doing so than are residential care providers. It was relatively easy to access |  |
| Country | **Policy & Regulation** | **Financing** | **Organisation & Service delivery** | **Clients & Informal care givers** |
| **UK cont. III** | services is growing [67]. Still, there is little national regulation on domiciliary care in England [18]. The user experience surveys should contain at least 4 questions: on general satisfaction, whether social services check the user satisfaction, whether they are visited at suitable times and whether if they ask for a change in help they receive it. There is a ‘methodological’ controversy around this satisfaction survey as a means to measure outcomes of service delivery [69]. |  | training in that 80% of social workers (in residential and home care) said that when following training their work was taken over by colleagues, and that the costs of training were covered by the employer for 90% of the employees following training [71].  In England, care by independent providers is perceived of lower quality by older users compared with in-house providers [70]. In Northern Ireland, a statutory service seemed less likely to take responsibility for deciding on acceptability of working conditions in case of contracted private businesses . In case of provision by an in-house department the organisation had more written standards and procedures to regulate the service, and the managers were more likely to regard it as their responsibility to ensure a safe working environment [67]. |  |
| **Multiple country studies** | The elderly care sector in France, Germany and England was more or less shared between the public sector and the third sector, and hence a ‘network’ rather than monopoly existed [25]. A recent change in this sector has been creating a more ‘for-profit’ attitude through setting measurable outputs and comparing them with costs. Although still very important co-governance (setting regulation based on cooperation of the government, civic and professional actors) has been partially replaced by market forces. Market mechanisms have weakened and disorganised network relations based on consensus. However, there are some country differences regarding the influence of competition. In Germany, cooperation between providers and co-governance has slightly weakened by the introduction of competitive elements. | There are several ways of funding home care. In Germany, home care is financed through the long-term care insurance (administrated by subunits of the quasi-public health funds). In-kind benefits may be replaced with cash benefits [25] In France, care for the elderly persons is financed through the long-term care scheme granting an earmarked allowance. Client co-payments are required for those with a high income. However, the French counties may opt out of the funding regime [25].  Le Bihan et al. distinguish three ways of taking income into account in home care assignment: 1. family income, 2. the income of the dependent person is taken into account 3. the resources of the dependent person [23].  In the UK and Sweden means-tested | Taking similar levels of care dependency into account, countries in Europe extremely differed in the extent of formal care provided. In Italy, for example, very little formal care was available, while in England more than two times the median hours of all 11 countries was available across all levels of dependency [72].  Countries differ in the nature of home care providers. In 2006, in Germany there were several types of agencies providing home care: non-profit, for-profit and religion-based organisations [25]. They are competing for the same clients. Home care workers have become less professionalised due to a downward pressure on prices and hence time saving has become important [25]. The provision of home help in France is done by publicly funded, county licensed, non-profit organisations. Additionally, employment of private care workers by households has been encouraged by the government, and hence, organisations leasing private providers have grown in importance. Regional social service providers | Recipients of community care in France and Italy have high physical and cognitive impairment compared to northern Europe. [76]  In home care, formal and informal care can be substitutes, but this relationship differs across Europe. The substitution effect of informal care is significantly lower in Central Europe (Germany, France, Netherlands, Austria and Switzerland) than in Southern Europe (Spain, Italy and Greece). In Central Europe, there is hardly any effect of informal care on the provision of formal home care. This is also true if controlling for the endogeneity of informal care [73].  In the Netherlands, Germany and Austria, clients had experienced more choice through the cash programmes [22]. |
| Country | **Policy & Regulation** | **Financing** | **Organisation & Service delivery** | **Clients & Informal care givers** |
| **Multiple country studies cont.** | However, new non-profit alliances have been created through this and meetings between providers and public organisations on national and local level are being held. In France, there is still a strong public responsibility and hence more uniform standards and state control, but some initiatives created more competition. Furthermore, particularistic ‘local’ (clientele focused) agreements are being made. These result in disparities across the country. Personal care is less regulated than home nursing, Still the voluntary sector providing personal care and the public authority are involved in a cooperative network, for instance creating consensus decisions on training policies and on salaries.  In England, market forces (a long tradition) and public control of quality of social care are complemented by a voluntary sector and hence by co-governance [25]. Although non-profit organisations have to act in a more businesslike way, they are advocating and achieving more market regulation (such as regulation on quality and single assessment). Furthermore, infrastructural issues are dealt with through Local Strategic Partnership Boards and, sometimes, meetings between local authority and representatives of providers [25].  A current trend in Europe and the United States are care models combining consumer directed | co-payments based on the costs of care packages are required. In Barcelona (not nationally decided) and Rome there are even income thresholds. In France, low income recipients are not required to pay for their services [23]. Cash benefits to pay for care services are available in France, Germany, Sweden, UK, Italy, Spain [23], and Austria [22], and also cash-for-care programmes in Finland, the Netherlands and Ireland [24]. In Ireland, England, Finland and the Netherlands the arrangement was optional and were mainly financing private providers and self-employed individuals. In most of them the arrangement covered less services (/costs) than direct care (although differences in level). The reasons behind cash-for-care programmes are the same across Europe, but their relative weight differs across countries. Reasons are reducing institutional care to decrease costs (Ireland), to increase freedom of choice and decrease the role of traditional (inflexible) service care provision (in the England and the Netherlands) and stimulating private sector provision to substitute the decreasing public provision (Finland) [24]. The eligibility criteria for the arrangements also differ. While in Austria and Germany the benefits were not means-tested, they were in the Netherlands [22]. Additionally, | tended to opt for these firms. However, now the earmarked allowance scheme favours professional providers over these. In France, private firms are still scarce.  In France, home nursing is provided by national health insurance funded independent or municipal nurses. Some municipalities have set up integrated service centres providing both personal care and home nursing [25].  In England provision mainly takes place through independent providers (just a small share is voluntary) which are contracted through tenders by public bodies. Both organisations have become increasingly influenced by new public management (target setting, benchmarks etc.) [25].  Case managers in home care were unavailable in the Czech Republic, Denmark, France, Germany, the Netherlands and Norway [76].  In Germany, although municipal advice centres and administrative bodies of the insurance (needs assessors) inform clients on the available services, there is no central gatekeeper able to inform clients on all services available [25].  In many countries (all countries studied by Bihan et al. [23]) there is a personal needs assessment, performed by a team of professionals (doctors and social workers) who visit the persons to assess the ADL needs, their physical and psychological pathologies and their social and familial environment [23]. In France, Germany, the UK, Sweden and Spain, the type of services and the number of hours are determined based on the assessment, and laid down in a care plan [23]. Even so, countries differ in their needs assessment regarding the instruments used, the professionals performing it and whether the assessment is social or medical of nature. The assessment is more formalised (national scale) in France than in the UK and Sweden where |  |
| Country | **Policy & Regulation** | **Financing** | **Organisation & Service delivery** | **Clients & Informal care givers** |
| **Multiple country studies cont. 2** | approaches (as a reaction to managerial approaches), service packages managed by providers and professionals, and pooled funding [22]. Consumer movements have pushed for more self-determination, but also for de-institutionalisation. Three main approaches to consumer directed care can be distinguished: the professionally monitored model (clients have the choice of provider), the professionally assisted model (clients additionally decide on scheduling and are responsible for supervision) and the cash model (complete direction given by clients) [22].  In 2003, care for the elderly persons is managed at local level in many countries, i.e. in the UK, Sweden, Italy, Germany, France and Spain. Still, in all these countries the provision of domiciliary care for frail elderly people is a policy priority [23]. | countries differed in the quality control over services paid through the cash arrangements (high level of control in NL and FI; less in the UK and a very low level in IR), in whether informal caregivers could be paid (in NL and UK they were, in IR and FI they were not) and the level of cost coverage of direct care (lowest in IR, 50% in DE [22], 75% in NL and highest in Finland) [24].  The way in which the level of cash benefit is decided also differs across countries. In Sweden, it is part of the overall needs assessment. In Germany, Spain, Italy, France and the UK, the decision on the care package follows a separate procedure from the needs assessment [23].  The freedom to decide what to spend the cash on differs between and inside countries (e.g. in Ireland). In some areas recipients pay and employ care providers themselves, in others, the care providers are paid directly after the services are delivered. In England, ‘Direct Payments’ (DP) are complementary to other health and social services, as in Ireland [24].  Problems arising in cash-for-care programmes are e.g. the lack of regulation (Ireland) [24], coverage of costs (England and Ireland) [24], insufficient mechanisms to support those eligible in making a choice [22] (Germany) and barriers to take up the arrangement, such as lack of | there is a large discretionary power of the assessors. In Italy and Spain, the assessment may vary per region and assessment team. In these countries, allocated services depend on the available resources..In Barcelona, an increase in dependency is not always associated with an increase in service provision. In the other countries studied the correlation was usually positive. In the French Ille-et-Vilaine, next to an increase in hours this also leads to an increasing urgency of arranging (quasi) institutional settings.  In Sweden, the link is not direct: care managers decide on the intervals and frequency of care based on the loss of autonomy, the type of care required and number of hours needed. However, currently, procedures are based on international standards and not only left to the experience of care managers. In Germany, there is a direct link between the care need and the type of care.  In the UK, local authority social service departments decide upon the care plan (which is changed every 6 months) and case managers of social service departments inform clients on DP. In Finland, the needs are assessed (every six months) by a municipal care team. This team also informs clients about the availability of the vouchers. In the Netherlands, needs are assessed (by independent assessment agencies) and after this the client can opt for either direct provision or a personal budget [24].  A case study was performed in 6 countries comparing the assigned care packages. Although all the studied countries did have the same services available the level of service delivery in terms of hours and eligibility differed across countries. Alarm systems were scarcely available in the Spanish and Italian |  |
| Country | **Policy & Regulation** | **Financing** | **Organisation & Service delivery** | **Clients & Informal care givers** |
| **Multiple country studies cont. 3** |  | knowledge on the availability of the payment, professionals wanting to keep control over funding, bureaucracy, opposition by some local authorities and public trade unions (England), and the inability of persons with a cognitive impairment to use them (Finland) [24].  Although the cash-for-care programmes’ elements fit in with the overall care system in that country, there are some steps away from their traditional way of organising community care. In Ireland this is expected to increase private provision and funding [24]. | study site [23]. Institutional care was more likely to be granted in Italy and Spain [23] (as maximum hours to be provided at home were in 2006 3 and 11 hours a week). Countries differ in whether ‘care packages’ include both health and social services (in the UK and Italy they do and in Sweden and Spain only personal care services are included in care packages). In the UK, Spanish and Italian site a combination of day care and home care is regularly recommended in case of Alzheimer. In this case the care indicated is laid down very detailed in the UK unlike in the other countries. In Sweden care is assigned only for a few months as to have regular monitoring of care [23].  Problems in the care quality varied substantially between various sites in different countries. No country-specific site consistently scored worst or best. Unwanted outcomes were most prevalent in the Czech Republic, Italy, and Germany [10]. Common problems on quality of home care across Europe are rehabilitation potential in ADL; lack of therapy and inadequate pain control [10]. |  |
